# A New Paleozoic Symmoriiformes (Chondrichthyes) from the Late Carboniferous of Kansas (USA) and Cladistic Analysis of Early Chondrichthyans

**DOI:** 10.1371/journal.pone.0024938

**Published:** 2011-09-27

**Authors:** Alan Pradel, Paul Tafforeau, John G. Maisey, Philippe Janvier

**Affiliations:** 1 Department of Vertebrate Paleontology, American Museum of Natural History, New York, New York, United States of America; 2 European Synchrotron Radiation Facility, Grenoble, France; 3 Muséum National d'Histoire Naturelle, Centre de Recherches sur la Paléobiodiversité et les Paléoenvironnements (CRP2), UMR 7207 du CNRS, Département Histoire de la Terre, Paris, France; University of Maryland, United States of America

## Abstract

**Background:**

The relationships of cartilaginous fishes are discussed in the light of well preserved three-dimensional Paleozoic specimens. There is no consensus to date on the interrelationship of Paleozoic chondrichthyans, although three main phylogenetic hypotheses exist in the current literature: 1. the Paleozoic shark-like chondrichthyans, such as the Symmoriiformes, are grouped along with the modern sharks (neoselachians) into a clade which is sister group of holocephalans; 2. the Symmoriiformes are related to holocephalans, whereas the other Paleozoic shark-like chondrichthyans are related to neoselachians; 3. many Paleozoic shark-like chondrichthyans, such as the Symmoriiformes, are stem chondrichthyans, whereas stem and crown holocephalans are sister group to the stem and crown neoselachians in a crown-chondrichthyan clade. This third hypothesis was proposed recently, based mainly on dental characters.

**Methodology/Principal Findings:**

On the basis of two well preserved chondrichthyan neurocrania from the Late Carboniferous of Kansas, USA, we describe here a new species of Symmoriiformes, *Kawichthys moodiei* gen. et sp. nov., which was investigated by means of computerized X-ray synchrotron microtomography. We present a new phylogenetic analysis based on neurocranial characters, which supports the third hypothesis and corroborates the hypothesis that crown-group chondrichthyans (Holocephali+Neoselachii) form a tightly-knit group within the chondrichthyan total group, by providing additional, non dental characters.

**Conclusions/Significance:**

Our results highlight the importance of new well preserved Paleozoic fossils and new techniques of observation, and suggest that a new look at the synapomorphies of the crown-group chondrichthyans would be worthwhile in terms of understanding the adaptive significance of phylogenetically important characters.

## Introduction

Recent cartilaginous fishes, or chondrichthyans, comprise two clades, the sharks and rays (Neoselachii), and the chimaeroids (Chimaeriformes). Interrelationships of Paleozoic chondrichthyans to these clades are controversial, and will be re-examined here in the light of new three dimensionally preserved specimens and a new method of observation, the computerized X-ray synchrotron microtomography (SR-μCT Scan). Computerized X-ray microtomography scanning technology have been successfully applied to some preserved fossil chondrichthyan neurocrania and have provided valuable information on external and internal anatomical cranial features for comparative and evolutionary studies. Nevertheless, only a few examples have been studied so far by this way to date: *Cladodoides wildungensis* [1], a symmoriiform, “*Cobelodus*” [2], the hybodonts *Tribodus limae* [3] and *Egertonodus basanus* [3], [4], the iniopterygian *Iniopera* sp. [5]–[7], and in less detail *Pucapampella* [4], *Doliodus* [8] and *Acronemus* [9]. In addition, only a few cladistic studies based on early chondrichthyans exist in the literature, and the interrelationships of most Paleozoic chondrichthyans are still largely unresolved. At present, there are three main competing phylogenetic hypotheses: (a) all the Paleozoic chondrichthyans with a shark-like gestalt (such as the xenacanths, “ctenacanths”, symmoriiforms) are related to the Euselachii (Hybodontiformes plus stem and crown-Neoselachii) [10]–[14], and this clade (sometimes termed “Elasmobranchii”, although such usage is probably unjustified) is sister group to the Euchondrocephali, which include the stem holocephalans plus stem and crown-group chimaeriforms; (b) symmoriiforms are stem-group holocephalans, whereas the xenacanths and “ctenacanths” are related to the Euselachii [15]–[17]; and (c) the Euchondrocephali are a monophyletic sister group of the Euselachii in a crown-group chondrichthyans, whereas many Paleozoic “sharks” emerge as stem-chondrichthyans [18]–[20].

The Upper Carboniferous (Pennsylvanian) of Kansas, USA, has provided some three-dimensionally preserved chondrichthyan braincases belonging to the Sibyrhynchidae, one of two iniopterygian families currently recognized. Iniopterygians are supposedly related to extant chimaeroids [5]–[7]. Two other three-dimensionally preserved chondrichthyan neurocrania, both of which can be referred to a new genus, occur in the same locality as the sibyrhynchid braincases and are described in the present work. Computerized X-ray synchrotron microtomography reveals that their anatomy differs strongly from that of the sibyrhynchids. Their overall dorsal morphology recalls another poorly known iniopterygian representative, *Cervifurca nasuta*, which is referred to the other iniopterygian family, the Iniopterygidae [21]. Nevertheless, some characters (notably those concerning the basicranium, a probable interorbital septum and the cranial ornamentation) are in fact quite similar to those of some symmoriiforms (e.g., “*Cobelodus*” [2]; *Stethacanthulus meccaensis* [22], [23]; *Akmonistion* [24]). Interrelationships of early chondrichthyans, especially concerning the iniopterygians and symmoriiforms are still discussed and there is no consensus to date [2], [8], [14]–[17], [24], [25].

A description of this new material from the Pennsylvanian of Kansas is provided here, with a cladistic analysis of early chondrichthyans based on neurocranial characters.

## Materials and Methods

### Materials

**Figure 1:**
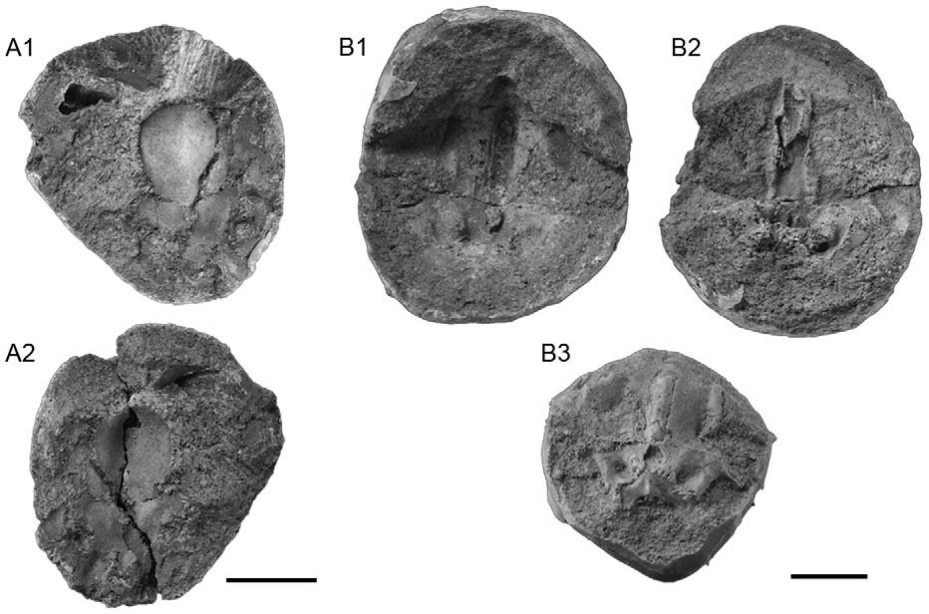
Photograph of the different nodules. A, part (A1) and counterpart (A2) of KUVP 152144. B, part (B1), counterpart (B2) and silicone cast of the part (B3) of KUVP 56340. Scale bar = 1 cm.

The material under investigation belongs to the Kansas University Natural History Museum, Lawrence USA, loaned by Dr. Larry Martin (Curator in Charge, KUNHM). It consists in two three-dimensionally preserved braincases (KUVP 152144, KUVP 56340) occasionally associated with some postcranial elements. These elements are preserved inside ovoid phosphatic nodules, the size of which varies from three cm to five cm in width and height (Fig. 1).

### Methods

#### Scanning

The specimens were imaged using X-ray based synchrotron microtomography (SR-μCT) [26] with absorption contrast on beamline ID19 of the European Synchrotron Radiation Facility, Grenoble, France. Scan parameters were as follow: monochromatic X-ray beam of 60 keV energy. The detector was a FReLoN (Fast Readout Low Noise) [27] CCD camera coupled with an optical magnification system, yielding an isotropic pixel size of 30.3 µm. 1200/180° projections with 0.4 s of exposure time. Data were reconstructed using the filtered backprojection algorithm (PyHST software, ESRF). Reconstructed slices were converted from 32 bits to 8 bits in order to reduce the data size for 3D processing.

Segmentation and 3D rendering were performed with MIMICS 64 bits version software (® Materialise Inc. NV, Leuven, Belgium).

#### Phylogenetic analysis

The data matrix was constructed in Mesquite [28] and processed using a heuristic search method: Traditional Search of TNT [29]. We also used TNT to run Bremer Support. Character states were treated as unordered (non-additive). The character state changes were visualized on the tree by using Winclada [30].

### Nomenclatural Acts

The electronic version of this document does not represent a published work according to the International Code of Zoological Nomenclature (ICZN), and hence the nomenclatural acts contained in the electronic version are not available under that Code from the electronic edition. Therefore, a separate edition of this document was produced by a method that assures numerous identical and durable copies, and those copies were simultaneously obtainable (from the publication date noted on the first page of this article) for the purpose of providing a public and permanent scientific record, in accordance with Article 8.1 of the Code. The separate print-only edition is available on request from PLoS by sending a request to PLoS ONE, Public Library of Science, 1160 Battery Street, Suite 100, San Francisco, CA 94111, USA along with a check for $10 (to cover printing and postage) payable to “Public Library of Science”.

In addition, this published work and the nomenclatural acts it contains have been registered in ZooBank, the proposed online registration system for the ICZN. The ZooBank LSIDs (Life Science Identifiers) can be resolved and the associated information viewed through any standard web browser by appending the LSID to the prefix “http://zoobank.org/”. The LSID for this publication is: urn:lsid:zoobank.org:pub:4581E1AC-0089-4241-9CAA-EB7BC2107183.

## Results

### Systematic paleontology

Class CHONDRYCHTHYES Huxley, 1880

Order SYMMORIIFORMES Zangerl, 1981

Genus KAWICHTHYS, gen. nov.

urn:lsid:zoobank.org:act:03A96D3D-A8F6-4109-B255-B686CBE3DF9A

#### Remarks

The systematics of the symmoriiforms is still in disarray [31]. For the purposes of the present work, the Symmoriiformes will be treated as monophyletic, despite indication from recent papers that these taxa may be paraphyletic [16], [17].

#### Type Species

*Kawichthys moodiei*, sp. nov.

#### Diagnosis

According to the present description and phylogenetic analysis, *Kawichthys* may be diagnosed as follows:

Symplesiomorphies at various levels: tesserate prismatic calcified cartilage; persistent otico-occipital fissure; endolymphatic fossa divided from fissure by a posterior tectum; postorbital plus palatobasal palatoquadrate articulations; lateral dorsal aortae contained by basicranial canals; chondrified lateral commissure enclosing the jugular canal; crus commune between the anterior and posterior semicircular canals; unchondrified medial capsular wall.

Possible synapomorphies with symmoriiforms: narrow suborbital shelf; Y-shaped division of dorsal aorta into paired lateral dorsal aortae at the level of the occipital region of the braincase, with lateral dorsal aortae running into basicranial canals; tropibasic skull; single-crowned, non-growing, and posteriorly directed cranial cap scales.

Possible synapomorphies with some symmoriiforms (e.g., *Akmonistion*, *Stethacanthulus*): narrow interorbital floor; broad supraorbital shelf which exceeds the lateral margin of the suborbital shelf; distinct hyoidean artery foramina.

Autapomorphies of genus and species: supraorbital shelf pierced by a large fenestra; internal carotid arteries running entirely within basicanial canals; apical extremity of the roof of the braincase overhung by a median dorsal crest, which bears posteriorly directed sharp denticles that are arranged symmetrically (from anterior to posterior, a median denticle is sandwiched by a pair of lateral denticles, except in the posteriormost part of the crest where two lateral pairs of denticles in suite are followed by two median denticles in suite).

#### Etymology

from “kaw” (a native American tribe from Kansas, whose men shaved their heads, leaving only the scalp lock uncut as a crest -www.kawnation.com-) and “ichthys” (Greek for “fish”).

*Kawichthys moodiei*, sp. nov.

urn:lsid:zoobank.org:act:7A33B33F-59E5-451A-966A-F8AC8B382B45

#### Holotype

KUVP 152144 (Figs. 1A1, A2). The specimen is a neurocranium associated with some disturbed and broken postcranial elements. The braincase is dorsoventrally crushed, so that its original depth is unknown. The orbital cartilages, almost all the ethmoid region, part of the supraorbital shelf and the occipital arch is missing, as well as the internal part of the braincase except for part of the endocranial cavity at the level of the inferred embryonic parachordal plate, the inferred embryonic trabecular/polar plate and the dorsal part of the orbital region. The basicranium is well preserved but the posterodorsal part of the skull is damaged.

#### Referred specimens

KUVP 56340 (Figs. 1B1–B3). This specimen is a neurocranium, which is well preserved in three dimensions, but lacks part of the ethmoid region, the occipital arch, the postorbital processes, the anterior part of the endocranial cavity, the orbital cartilages and probably the distal extremity of the median head crest (the cranial cap scales are not preserved). Nevertheless, the posterior part of the endocranial cavity and part of the skeletal labyrinth are present. The original cartilage is not preserved in most of the skull, so that only the cast of the external surface of the braincase could be used for the 3D reconstruction.

#### Remarks

**Figure 2:**
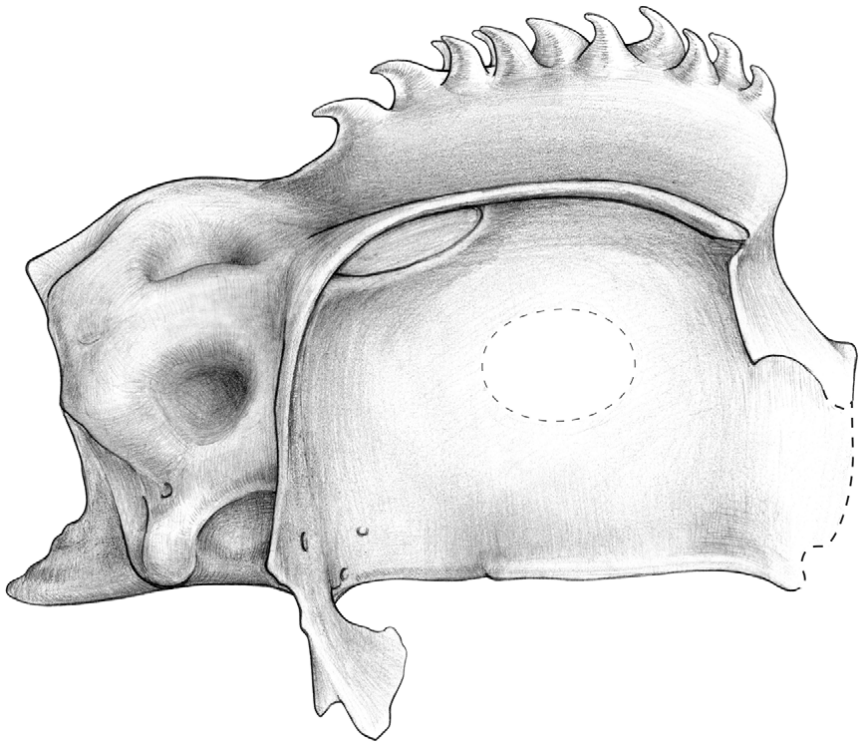
Attempted composite reconstruction of the braincase of *Kawichthys* in right lateral view based on the different 3D reconstructions. Not to scale.

KUVP 152144 and KUVP 56340 have the same dimensions (about 2.4 cm in length) and the overall morphology and the arrangement of the different structures of the neurocranium are similar in both specimens. The lack of certain features may be due to differences in preservation, since KUVP 152144 is dorsoventrally crushed and broken, and/or may represent intraspecific variability. The following description is thus based on both specimens, depending on their best preserved parts respectively. A drawing of the chondrocranium of an idealized *Kawichthys* specimen, which combines the morphology of the two specimens studied here, is shown in Figure 2. KUVP 152144 makes the holotype despite its crushing because it retains more diagnostic characters.

#### Locality and horizon

The specimens come from an outcrop approximately 150 km southeast of Kansas City, between Lawrence and Baldwin. Stratigraphically, it occurs at the limit between the marine Haskell Limestone Member and the overlying Robbins Shale of the Stranger Formation, which is interpreted as having formed in brackish water (see [6] for details on the paleoenvironment and the taphonomy). This sequence is part of the Douglas Group, dated as Late Virgilian, Upper Pennsylvanian (Ca 305-299 Myr).

#### Etymology

The species is named “moodiei” after Roy L. Moodie, who first studied the vertebrate remains of Kansas Paleozoic fish localities during the beginning of the 20^th^ century.

### Description of the braincase

#### Ethmoid region

**Figure 3:**
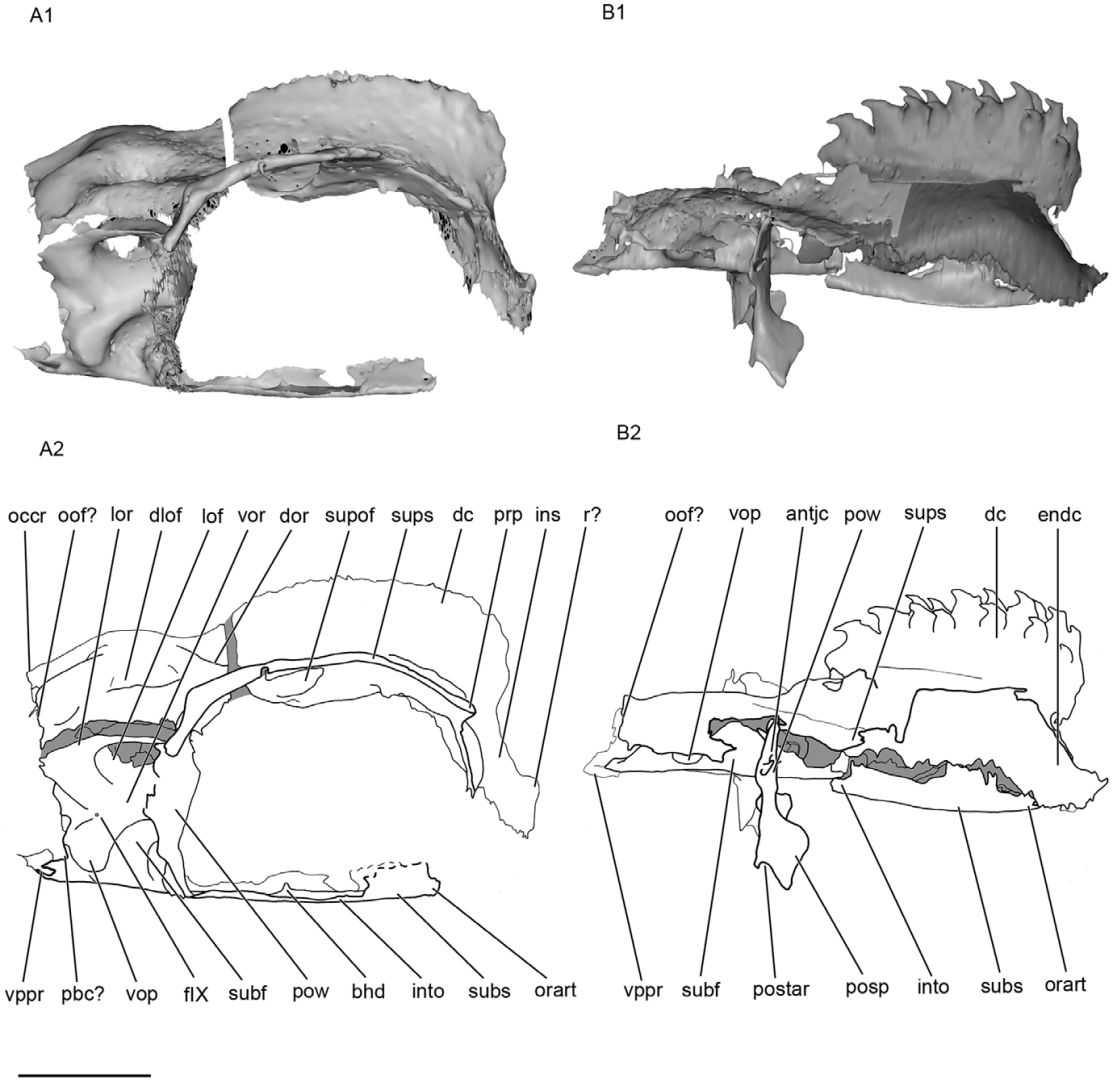
Lateral view of the skull of *Kawichthys*. Right side. A1, surface rendering generated from Synchrotron Radiation X-ray microtomographic slices of KUVP 56340. Part of the dorsal surface of the interorbital plate removed (dotted line). A2, corresponding drawing. B1, surface rendering generated from Synchrotron slices KUVP 152144. Dark grey, internal surface of the endocranial cavity and dermal median dorsal crest; light grey, external surface of the braincase. B2, corresponding drawing. Scale bar = 0.5 cm. Abbreviations: antjc, anterior opening of the jugular canal; bhd, bucco-hypophyseal duct; dc, dorsal crest; dlof, dorsolateral otic fossa; dor, dorsal otic ridge; endc, endocranial cavity; fIX, foramen for the glossopharygeus nerve; ins, internasal septum; into; interorbital plate; lof, lateral otic fossa; lor, lateral otic ridge; orart, orbital articulation for the palatoquadrate; occr, occipital crest; oof?, otico-occipital fissure; pbc?, posterior basicapsular commissure; postar, postorbital articulation for palatoquadrate; postp, postorbital process; pow, postorbital wall; prp, preorbital process; r?, rostrum; subf, subotic fossa; subs, suborbital shelf; supof, suborbital fenestra; sups, supraorbital shelf; vop, ventral otic process; vor, ventrolateral otic ridge; vppr, ventral paroccipital process.

Almost all the ethmoid region is missing in both specimens. Only part of the anterior median part of the roof of the neurocranium is present in KUNHN 56340. It strongly slopes ventrally to form a “bill”, and probably represents a vertical internasal septum (ins, Figs. 3A2; 4B2). The internasal septum extends slightly forwards, forming a small horizontal shelf that may represent part of a rostrum (r?, Figs. 3A2; 4B2; 5A2). In the corresponding region of KUVP 152144, the anterior extremity of the endocranial cavity displays the beginning of an anterior median horizontal opening, which may represent the posterior margin of a precerebral fontanelle (prcf?, Figs. 5B2; 6A2). If this is correct, the precerebral fontanelle was probably narrow, as in “*Cobelodus*” [2]. The internasal septum is quite broad posterodorsally and then tapers anteroventrally.

**Figure 4:**
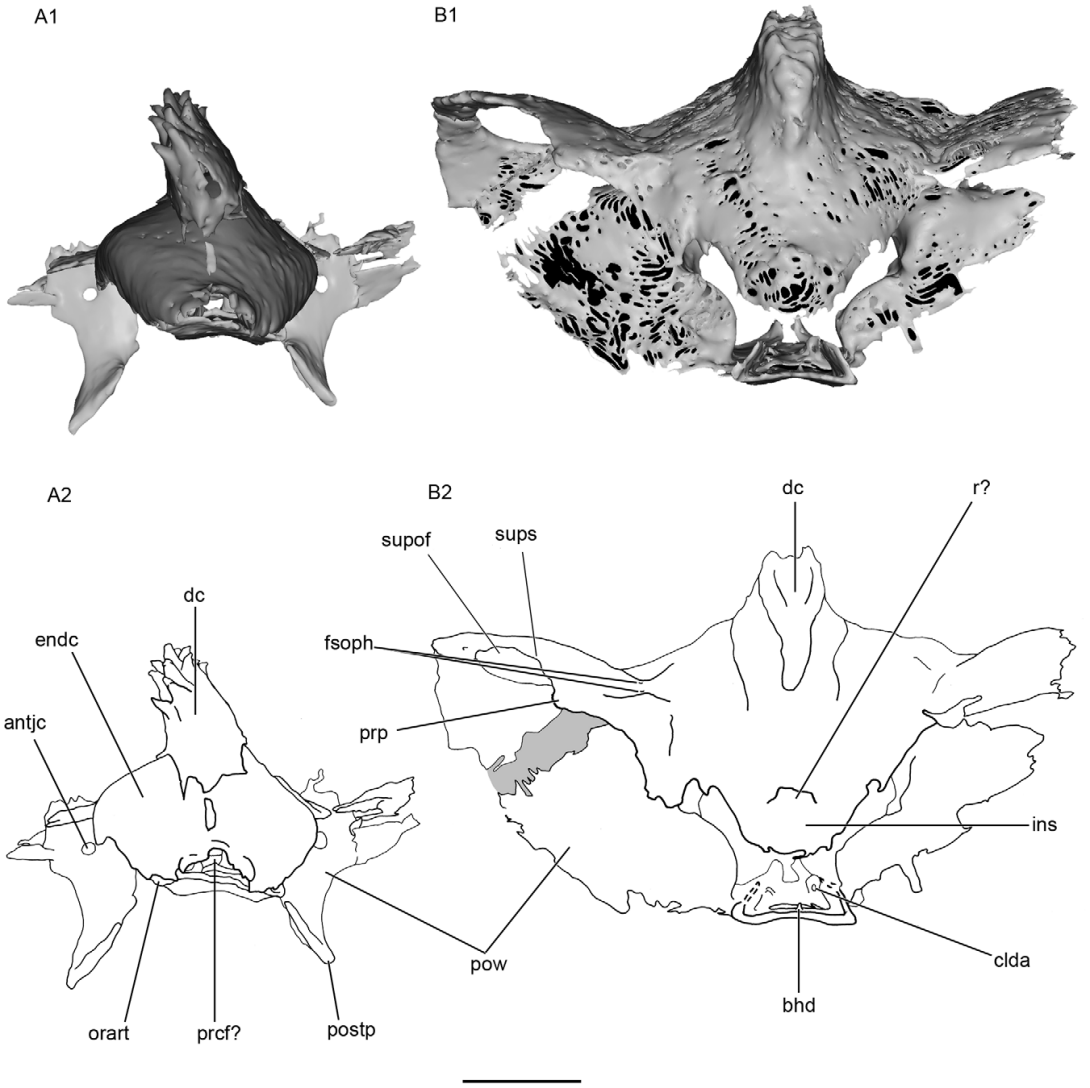
Anterior view of the skull of *Kawichthys*. A1, surface rendering generated from Synchrotron Radiation X-ray microtomographic slices of KUVP 152144. Dark grey, internal surface of the endocranial cavity and dermal median dorsal crest; light grey, external surface of the braincase. A2, corresponding drawing. B1, surface rendering generated from Synchrotron Radiation X-ray microtomographic slices of KUVP 56340. Part of the dorsal surface of the interorbital plate removed (dotted line). B2, corresponding drawing. Scale bar = 0.5. Abbreviations: clda, canal for lateral dorsal aorta; fsoph, foramen for the superficial ophthalmic nerve complex; prcf?, precerebral fontanelle. See Figs. 3 for other abbreviation.

**Figure 5:**
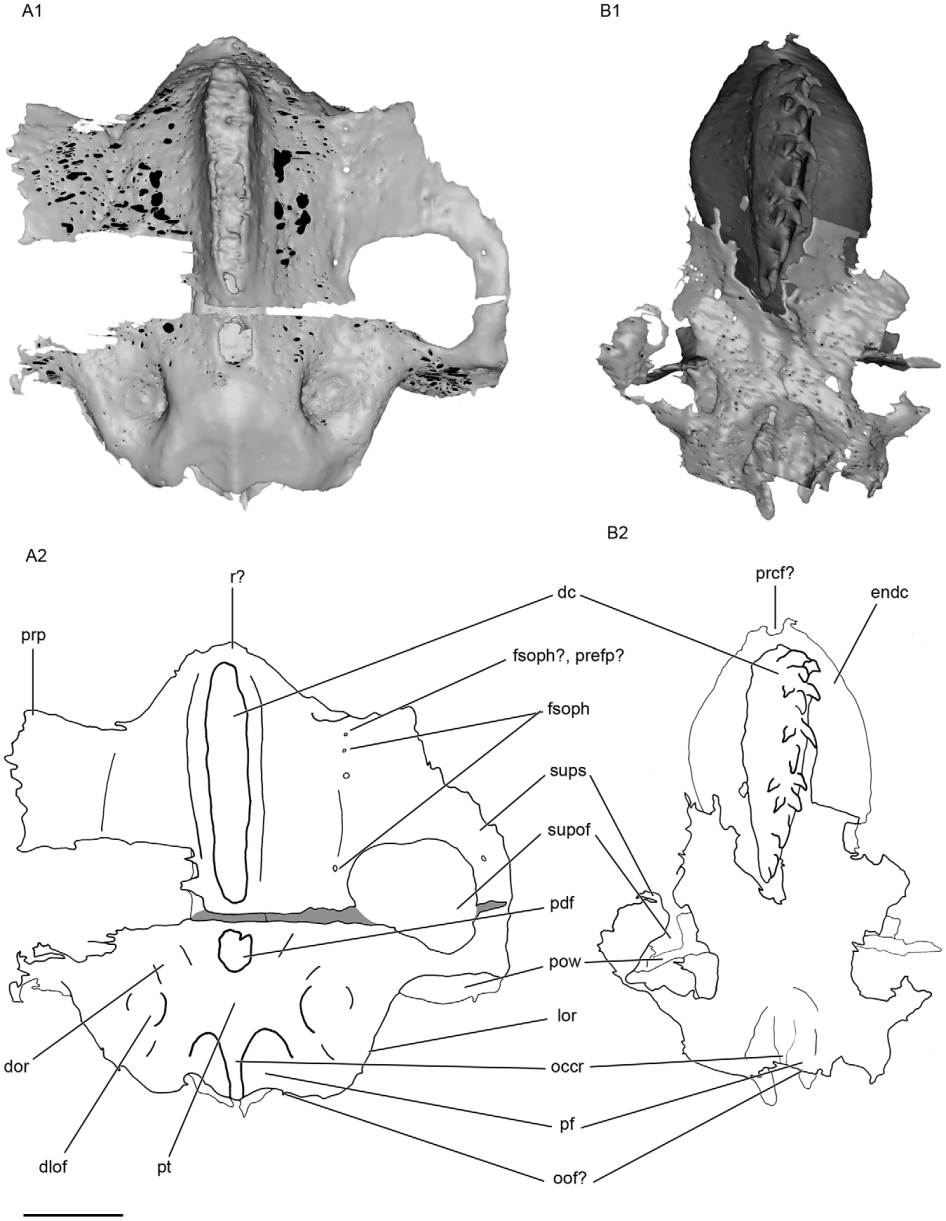
Dorsal view of the skull of *Kawichthys*. A1, surface rendering generated from Synchrotron Radiation X-ray microtomographic slices of KUVP 56340. A2, corresponding drawing. B1, surface rendering generated from Synchrotron Radiation X-ray microtomographic slices of KUVP 152144. Dark grey, internal surface of the endocranial cavity and dermal median dorsal crest; light grey, external surface of the braincase. B2, corresponding drawing. Scale bar = 0.5 cm. Abbreviations: pdf, posterior dorsal fontanelle; pf, preoccipital fossa; prefp?, preorbital foramen for the ophthalmic profundus ramus of the trigeminal nerve; pt, posterior tectum. See Figs. 3 and 4 for other abbreviation.

**Figure 6:**
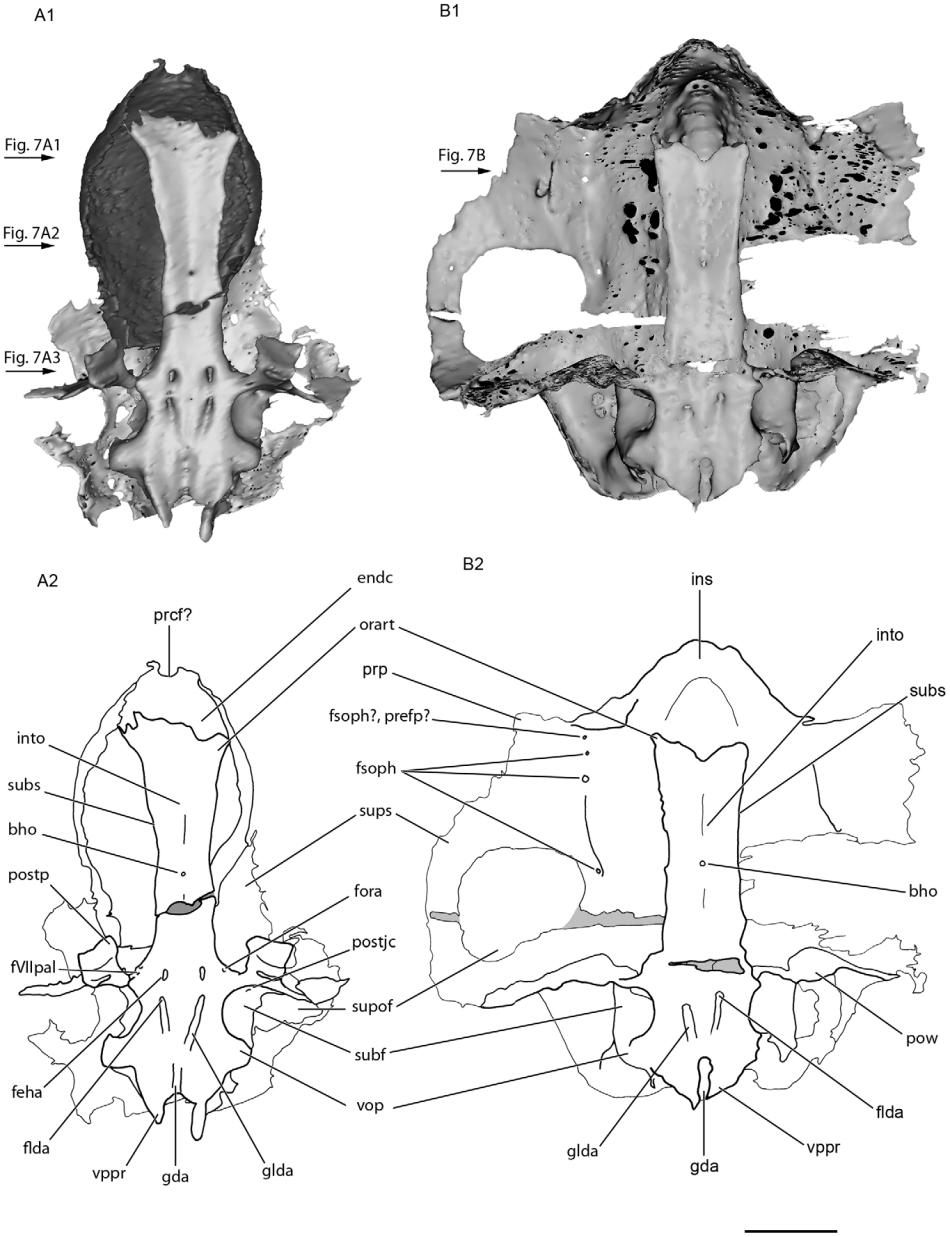
Ventral view of the skull of *Kawichthys*. A1, surface rendering generated from Synchrotron Radiation X-ray microtomographic slices of KUVP 152144. Dark grey, internal surface of the endocranial cavity; light grey, external surface of the braincase. A2, corresponding drawing. B1, surface rendering generated from Synchrotron Radiation microtomographic slices of KUVP 56340. B2, corresponding drawing. Scale bar = 0.5 cm. Abbreviations: bho, bucco-hypophyseal opening; feha, foramen for the efferent hyoidean artery; flda, foramen for lateral dorsal aorta; fora, foramen for the orbital artery; fVIIpal, foramen for the palatine ramus of facial nerve (undivided); gda, groove for the dorsal aorta; glda , groove for the lateral dorsal aorta; postjc, posterior opening of the jugular canal. See Figs. 3, 4 and 5 for other abbreviation.

#### Orbits

The orbital region comprises the region situated between the preorbital process anteriorly (prp, Figs. 3A2; 5A2), and the postorbital wall posteriorly (pow, Figs. 3A2; 5A2). It is delimited dorsally by the supraorbital shelf (sups, Figs. 3A2; 5A2).

The original depth of the orbit is not preserved in KUVP 152144, but can be estimated in KUVP 56340. Its orbits are remarkably large, almost as deep as long, and comprise more than half the length of the preserved skull.

#### Preorbital process and supraorbital shelf

The presumed embryonic limit between the supraorbital cartilage and the roof of the braincase is marked by a longitudinal groove, which is pierced by several foramina. The posteriormost ones are presumably for the passage of an ascending branch of the superficial ophthalmic complex (fsoph, Figs. 4B2; 5A2). A foramen is present at the anterior limit of this groove, but it is uncertain whether it also contained an ascending branch of the superficial ophthalmic complex, or possibly the ophthalmic profundus ramus of the trigeminal nerve (fsoph?, prefp?, Fig. 5A2).

From this groove, the supraorbital shelf flares dorsolaterally (sups, Fig. 4B2), so that the depth of the orbit appears identical to that of the braincase, and is wider than the suborbital shelf, as in some symmoriiforms, such as in *Stethacanthulus* [22] and *Akmonistion* [24]. The preorbital process is sharp and forms an almost right angle with the supraorbital shelf (prp, Figs. 3A2; 4B2; 5A2), as in *Cervifurca* [21]. It extends backwards slightly dorsolaterally and the supraorbital shelf flares laterally and forms a curved arcade that almost finishes right-angled with the postorbital wall (sups, pow, Figs. 3A2; 5A2). The rounded arcade delimits the lateral margin of a large fenestra (supof, Figs. 3A2; 4B2; 5A2), which is situated in the dorsal wall of the orbit. The lateral margin of these fenestrae is pierced by a foramen, but its function is hard to interpret. Similar fenestrae on the dorsal wall of the orbit are present in *Cervifurca* [21] and there is a deep notch in the corresponding part of the supraorbital shelf in *Stethacanthulus* and “*Cobelodus*” [2].

#### Median dorsal part of the orbital region and dorsal crest

**Figure 7:**
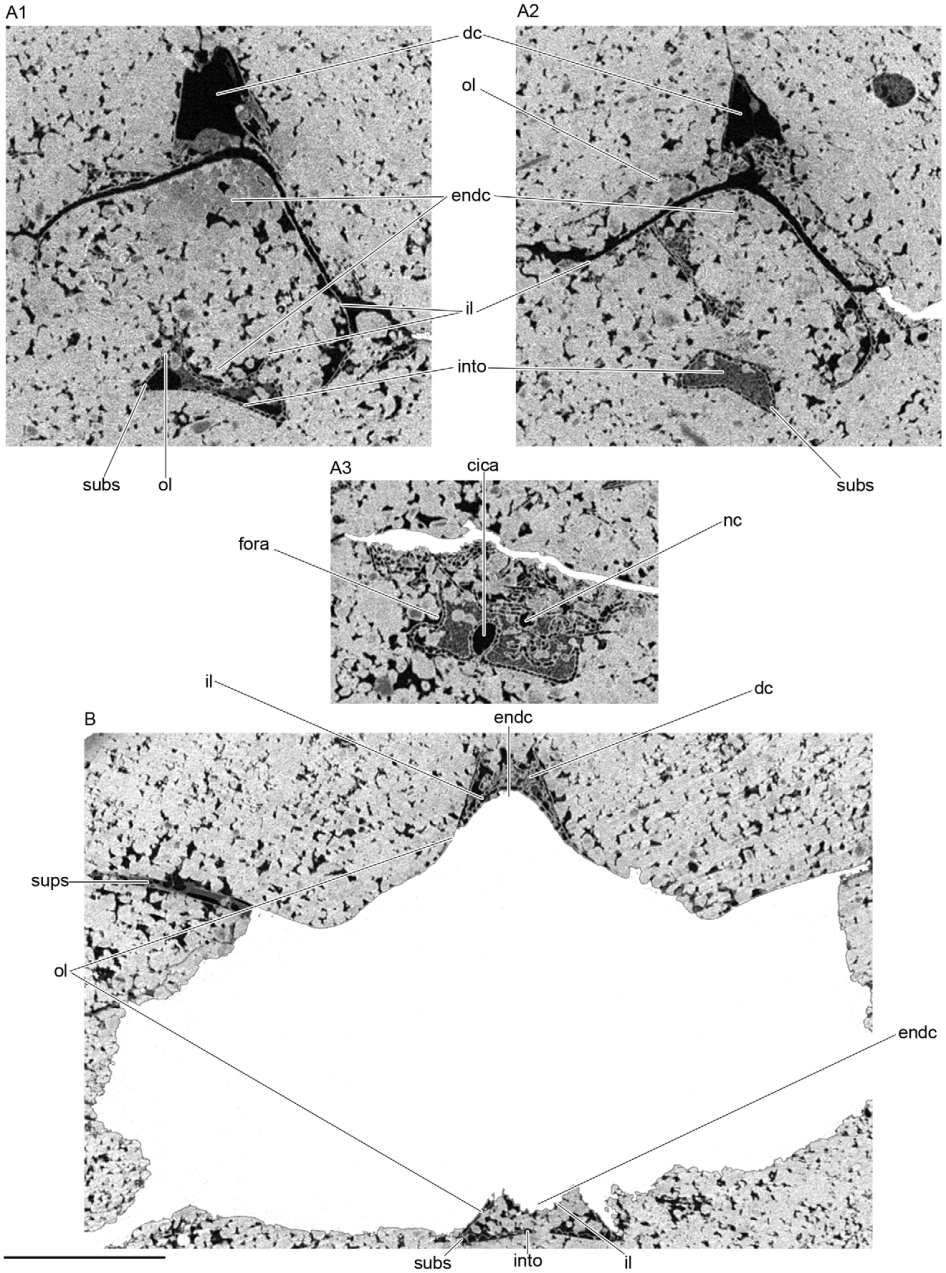
Transverse microtomographic slices through the interorbital plate of *Kawichthys*. Levels of the transverse slices are indicated by arrows in Fig. 6. A1, section through the anterior part of the interorbital plate and endocranial cavity of KUVP 152144. A2, section through the middle part of the interorbital plate of KUVP 152144, no ventral part of the endocranial cavity preserved . A3, section through the posterior part of the interorbital plate of KUVP 152144, no suborbital shelf preserved. B, section through the anterior part of the interorbital plate and endocranial cavity of KUVP 56340. Scale bar = 0.5 cm. Abbreviations: cica, canal for internal carotid artery; il, inner layer of the endocranium; nc, notochordal canal; ol, outer layer of the endocranium. See Figs. 3 and 6 for other abbreviation.

The apical extremity of the domed roof of the braincase in both KUVP 152144 and 56340 raises, forming a median dorsal crest that extends from the posterior dorsal fontanelle (dc, pdf, Fig. 5A2) to the posterior end of the internasal septum anteriorly. In KUVP 152144, this dome is situated below a crest of dermal denticles and lies below the external layer of cartilage of the skull. Consequently, in this specimen it probably corresponds to the inner layer of the braincase, i.e. the dorsal part of the endocranial cavity (dc, endc, Figs. 3B2; 4A2; 5B2; 7A1, A2). Although the external layer of the braincase of KUVP 152144 is not well preserved, the crest seems to be continuous with it, as in KUVP 56340 (dc, ol, Figs. 7A2, B). The dorsal crest is composed of small sharp denticles, which are all directed posteriorly and are arranged symmetrically: from anterior to posterior, a median denticle is sandwiched by a pair of lateral denticles, except in the posteriormost part of the crest where two lateral pairs of denticles in suite are followed by two median denticles in suite (dc, Figs. 3B2; 5B2). This denticle arrangement differs from that in *Stethacanthulus* and *Akmonistion*, in which the denticles are all approximately the same size and are arranged in numerous rows [2], [16].

#### Interorbital plate and suborbital shelf

The floor of the orbital region is narrow (into, Figs. 6A2, B2), as in *Stethacanthulus* [20] and *Akmonistion* [24], but flares laterally in its anterior part, forming two lateral processes (orart, Figs. 6A2, B2).

**Figure 8:**
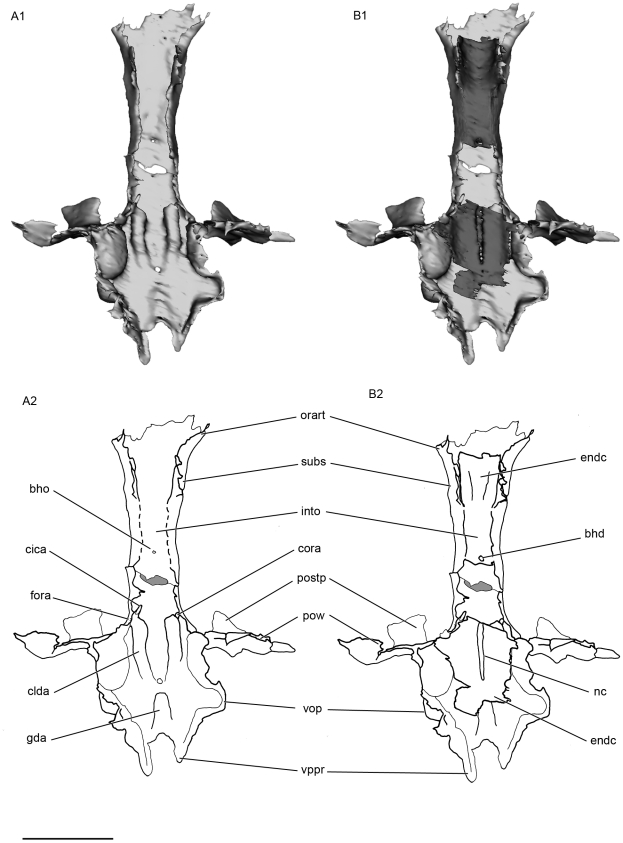
Dorsal view of the the basicranium of the skull of *Kawichthys* KUVP 152144. A1, surface rendering generated from Synchrotron Radiation X-ray microtomographic slices. Part of the dorsal surface of the interorbital plate removed (dotted line). Dark grey, internal surface of the floor of the endocranial cavity and interorbital plate; light grey, external surface of the braincase. A2, corresponding drawing. B1, surface rendering generated from Synchrotron Radiation X-ray microtomographic slices. Dark grey, internal surface of the endocranial cavity and interorbital plate; light grey, external surface of the braincase. B2, corresponding drawing. Scale bar = 0.5 cm. Abbreviations: cora, canal for the orbital artery. See Figs. 3, 4, 6 and 7 for other abbreviation.

The ventral surface of the interorbital plate is pierced medially by a single foramen, which probably represents the bucco-hypophyseal opening (bho, Figs. 6A2, B2). This is located farther anteriorly than in “*Cobelodus*” [2], *Akmonistion* [24] and *Cladoselache* [2]. In the mid region of the orbit, the interorbital plate is flat and is perfectly continuous dorsally and ventrally, and there is no separation between the outer and inner layers of prismatic calcified cartilage of the braincase (into, Figs. 7A2; 8A2, B2; 9A2, B2). A pair of foramina, situated at the level of the postorbital processes on the ventral surface of the KUVP 152144's basicranium (feha, Fig. 6A2), is connected to the canals for the lateral dorsal aortae (clda, Fig. 8A2). These paired foramina are absent in KUVP 56340, but this is certainly due to the lack of preserved cartilage because of a break at the level of the presumed position of these foramina.

**Figure 9:**
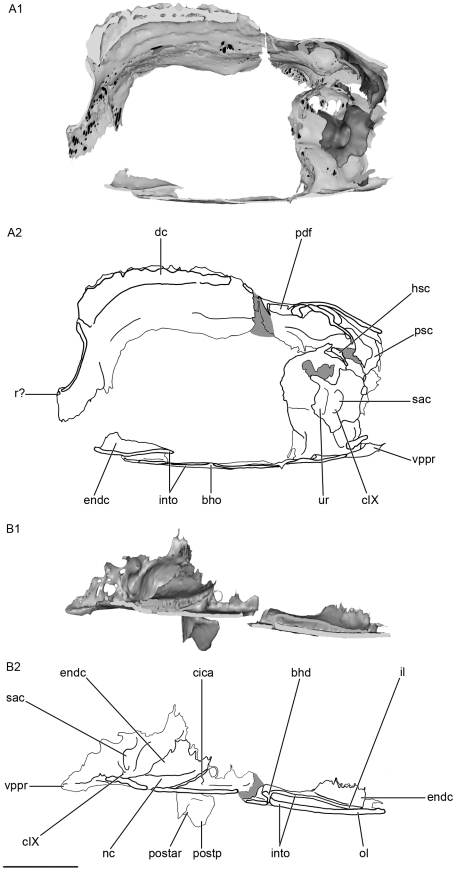
Medial view of the skull of *Kawichthys*. A1, sagittal section of surface rendering generated from Synchrotron Radiation X-ray microtomographic slices of KUVP 56340. Right side. Dark grey, internal surface of the endocranial and skeletal labyrinth cavity; light grey, external surface of the braincase. A2, corresponding drawing. B1, sagittal section of surface rendering generated from Synchrotron Radiation X-ray microtomographic slices of the basicranium and some preserved part of the endocranial and labyrinth cavity of KUVP 152144. Left side. Dark grey, internal surface of the endocranial cavity; light grey, external surface of the braincase. B2, corresponding drawing. Scale bar = 0.5 cm. Abbreviations: cIX, canal for the glossopharyngeus nerve; hsc, horizontal semicircular canal; psc, posterior semicircular canal; sac, saccular chamber; ur, utricular recess. See Figs. 3, 5, 6 and 7 for other abbreviation.

In cross section, the ventral surface of the interorbital plate flares slightly laterally to form a narrow, almost obsolete, suborbital shelf (subs, Figs 7A1, A2, B). It extends from the enlarged part of the interorbital plate to a point situated behind the level of the bucco-hypophyseal opening. The suborbital shelf extends farther anteriorly than in “*Cobelodus*”, as in *Akmonistion* and *Cladoselache*. But, contrary to most Paleozoic chondrichthyans, it is not continuous with the postorbital wall. The suborbital shelf is frequently narrow or absent in symmoriiforms (e.g., *Akmonistion* [24], *Falcatus* [32], *Damocles* [33], *Stethacanthulus* [22]).

#### Postorbital wall, postorbital process and jugular canal

As in “*Cobelodus*”, the supraorbital shelf merges posteriorly with a steep and thin postorbital wall (pow, Figs. 3A2, B2; 4A2, B2), which, in KUVP 152144, extends ventrally to form a thin postorbital process (the part of the postorbital wall that articulates with the palatoquadrate) (postp, Figs. 3B2; 4A2). KUVP 56340 shows no postorbital process below the postorbital wall, probably because of a break, since its postorbital wall is also not completely preserved. In dorsal view, the dorsal part of the postorbital wall is blunt and forms, with the supraorbital shelf, a right angle, lateral to the endolymphatic fossa (sups, pow, pdf, Fig. 5A2). Then, the postorbital wall is slightly rounded and extends far laterally, at the same level as the supraorbital shelf (pow, sups, Figs. 3A2; 4B2; 5A2). It is inferred that the embryonic lateral commissure is completely chondrified, since a complete chondrified postorbital wall is present, and the canal, which runs through the postorbital wall, probably represents the jugular canal (antjc, Figs. 3B2; 4A2; 10B; postjc, Figs. 6A2; 11B2). The jugular canal is extremely small in comparison with “*Cobelodus*”. The jugular canal is in a ventral position, relative to the basicranial floor. As in many Paleozoic chondrichthyans (except *Pucapampella* and “*Cobelodus*”) and neoselachians, the lateral commissure seems to have a ventral connection with the otic capsule. The postorbital wall is lateral to the anterior end of the skeletal labyrinth (ur, Fig. 9A2).

**Figure 10:**
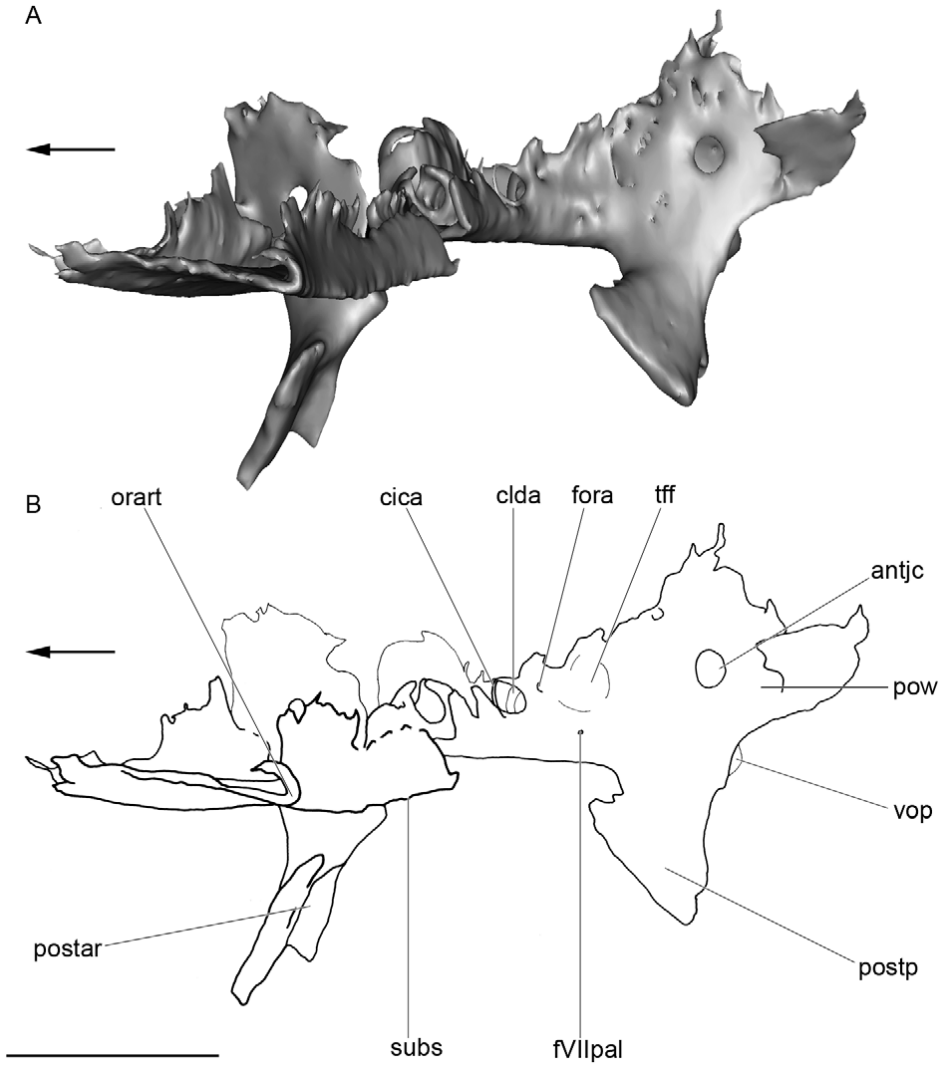
Oblique medial view of the basicranium of KUVP 152144. Part of the dorsal surface of the interorbital plate removed (dotted line). Left side. A, surface rendering generated from Synchrotron Radiation X-ray microtomographic slices. B, corresponding drawing. Arrows points forward. Scale bar = 0.5 cm. Abbreviations: tff, trigemino-facial fossa. See Figs. 3, 4, 6 and 7 for other abbreviation.

**Figure 11:**
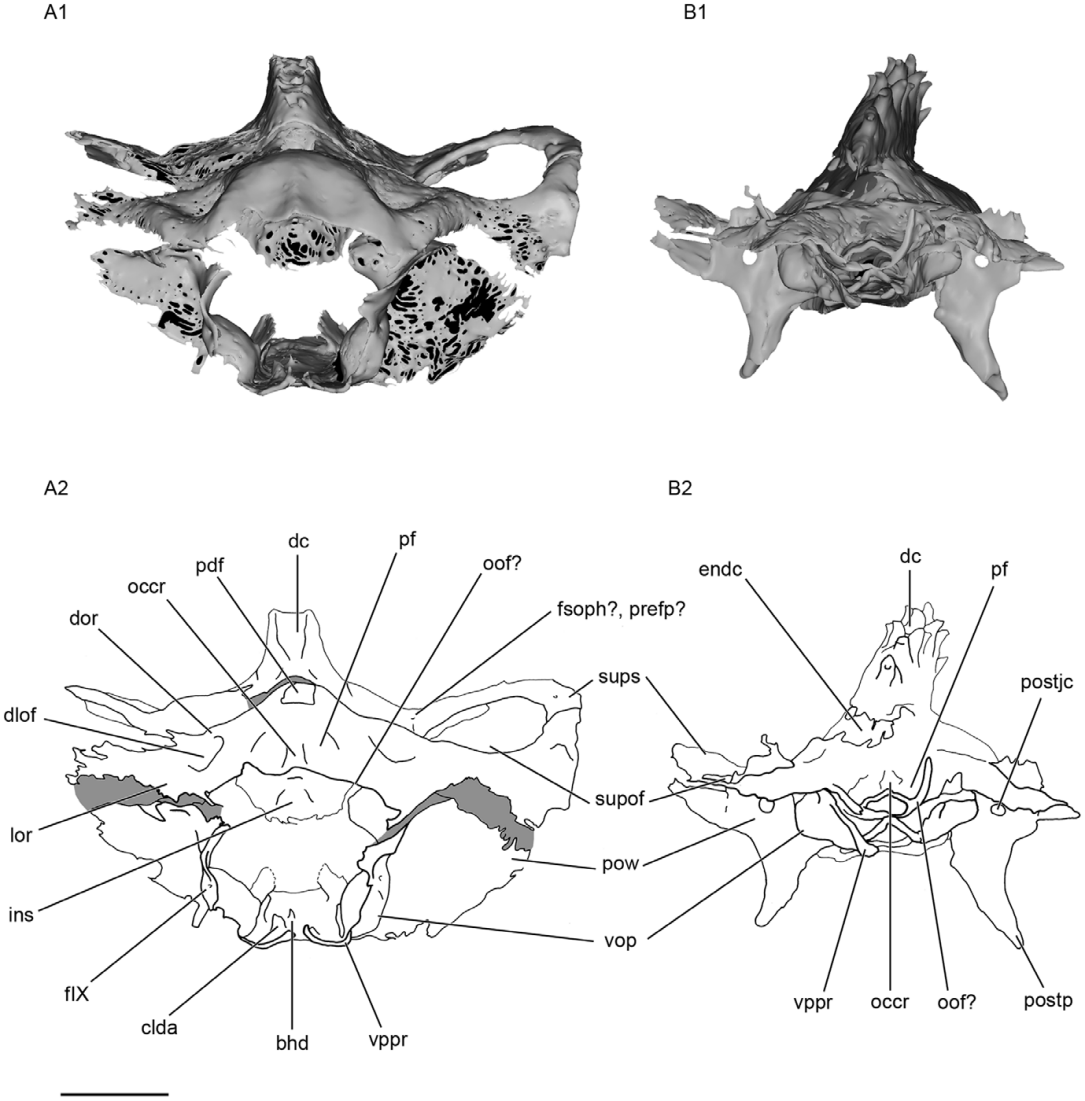
Posterior view of the skull of *Kawichthys*. A1, surface rendering generated from Synchrotron Radiation X-ray microtomographic slices of KUVP 56340 (A). A2, corresponding drawing. B1, surface rendering generated from Synchrotron Radiation X-ray microtomographic slices of KUVP 152144. Dark grey, internal surface of the endocranial cavity and dermal median dorsal crest; light grey, external surface of the braincase. B2, corresponding drawing. Scale bar = 0.5 cm. See Figs. 3, 4, 5 and 6 for the abbreviations.

The postorbital process extends farther ventrally than the surface of the basicranium plan (postp, subs, Fig. 3B2), as in *Tristychius* [34] and, to a lesser extent, in *Stethacanthulus* [22]. The distinction between the postorbital wall and the ventral postorbital process is well marked and is better observed in posterior view (pow, postp, Fig. 11B2): the postorbital wall shrinks ventrally below the jugular canal, so that the lateral border of the postorbital process does not extend as far laterally as the lateral margin of the postorbital wall. Consequently, the lateral margin of the postorbital process forms a concave surface in posterior view, and its dorsal part forms a process when it merges with the postorbital wall.

The articular facet of the postorbital process consists of a deep transverse groove, which is anteromedially directed, and flanked by two thin, but very well marked ridges (postar, Figs. 3B2; 9B2; 10B). The anteriomost one is curved, while the posteriormost is almost vertical and follows thereby the general inclination of the postorbital process. Similar overall morphology of the articular facet is also present in symmoriiforms. In *Cervifurca*, the condition is different: the articular facets for the palatoquadrate are not situated on a ventral postorbital process, but at the mid-height of the braincase, behind the orbit and lateral to the otic capsules [21].

The fact that the postorbital process extends far ventrally, and that it is curved compared to the postorbital wall, suggests that the palatoquadrate articulates with the postorbital process on its distal extremity, rather than on the posterior surface of the postorbital process, as in *Tristychius* [34] and some hybodonts.

The disposition of the articulation is similar to that of other Paleozoic chondrichthyans in which a chondrified lateral commissure is present, since it is situated on the latter and ventrolateral to the jugular canal. In addition, the orientation of the articular groove is the same as “*Cobelodus*” and *Cladodoides*.

#### Orbital artery, palatine nerve (VII) and trigemino-facial fossa

There is no evidence of foramina for the trigeminal nerve and the main branches of the facial nerve. Nevertheless, at the ventral junction between the postorbital wall and the floor of the braincase is a vertical canal, the anterior opening of which is situated in the posteromedial part of the orbit (fVIIpal, Figs. 6A2; 10B), and the posterior opening of which is located at the anterior end of the subotic fossa (subf, Figs. 3A2, B2). Consequently, the canal passes through the medial border of the postorbital wall, ventromedially to the jugular canal, and may represent a canal for the palatine ramus of the facial nerve. Dorsomedial to this foramen is another one, situated on the ventrolateral wall of the braincase, which corresponds to the opening of the canal for the orbital artery (fora, Figs. 6A2; 8A2; 10B).

The posterior part of the orbit contains a fossa located ventromedially on the lateral wall of the braincase (tff, Fig. 10B), anterior to the otic capsule. This fossa is delimited anteriorly by the orbital opening for the orbital artery (fora, Fig. 10B), ventrally by the orbital foramen for the palatine ramus of the facial nerve (fVIIpal, Fig. 10B), and laterally by the postorbital wall (pow, Fig. 10B). Unfortunately, its dorsal part is not preserved at this level. A fossa in this position is relatively common among chondrichthyans and represents either the trigemino-pituitary fossa *sensu* Allis [35], or a trigemino-facial fossa (which houses part of the ganglion of the two nerves, if they arose anterior to the postorbital wall, plus the external rectus musculature).

There is no evidence of foramina for either the abducens and trigeminal nerves, nor for the pituitary vein in the specimens studied here. Nevertheless, the bucco-hypophyseal opening is situated far anterior to this fossa, so that the pituitary vein was also probably far anterior to the fossa, since both mark the anterior limit of the inferred embryonic parachordal plate in chondrichthyans [36]. Consequently, the fossa probably does not represent a trigemino-pituitary fossa *sensu* Allis, and may either represent a trigemino-facial fossa, or a depression into which the external rectus musculature is inserted. A small fossa in “*Cobelodus*” also probably housed this musculature, and there is no trigemino-facialis fossa [2].

#### Otico-occipital region

The otic region is short, as in the Symmoriiformes generally. The posterior dorsal fontanelle appears in an anterior position (pdf, Fig. 5A2), wedged between the postorbital walls, immediately posterior to the median dorsal crest (dc, Fig. 5A2). The posterior dorsal fontanelle is rounded and there is neither evidence of endolymphatic foramina, nor perilymphatic fenestrae. However the endolymphatic ducts were probably present and passed from the posterior dorsal fontanelle through the unchondrified medial wall of the otic capsule (see below), as in extant chimaeroids [10], [37] and probably in *Cladodoides* [1], “*Cobelodus*” [2] and *Iniopera* sp. [6].

Paired dorsal otic ridges (dor, Fig. 5A2), which cover the dorsal part of the anterior semicircular canals, extend posteriorly from the medial margin of the posterior part of the suborbital fenestrae and converge on either sides of the posterior dorsal fontanelle posteriorly. They extend as far as the anterior margin of the occipital crest posteriorly (occr, Fig. 5A2). They are not strongly pronounced and do not form a horizontal crest on each side of the posterior dorsal fontanelle, contrary to the condition seen in *Orthacanthus*, *Tamiobatis* [10] and perhaps *Akmonistion* [24]. Instead, the low dorsal otic ridges merge posteriorly with the posterolaterally divergent ridges that cover the posterior semicircular canal. The meeting of the latter with the dorsal otic ridges delimits the medial border of relatively deep dorsal fossae (dlof, Fig. 5A2). The latter are bordered ventrolaterally by a lateral otic ridge (lor, Figs. 3A2; 5A2), which surrounds the horizontal semicircular canal, as in many Paleozoic sharks. This disposition suggests that the dorsal fossae may correspond to the dorsolateral otic fossae, which are present in many Paleozoic chondrichthyans. The occipital crest (occr, Figs. 5A2, B2) extends posteriorly and is flanked by the preoccipital fossae similar to those of many Paleozoic chondrichthyans (pf, Figs. 5A2, B2). The dorsolateral otic and preoccipital fossae and the dorsal otic ridges probably provided insertion areas for epaxial musculature.

The lateral otic ridge containing the horizontal semicircular canal overhangs another concave area (lof, Fig. 3A2), which has the same topographic position as the lateral otic fossa of many Paleozoic sharks, such as *Orthacanthus*, *Tamiobatis* [10], *Akmonistion* [24], *Cladodoides* [1] and “*Cobelodus*” [2]. The lateral otic fossa is separated from the subotic fossa (subf, Fig. 3B2) by a longitudinal ventrolateral otic ridge (vor, Fig. 3A2), which surrounds the saccular chamber and the utricular recess (sac, ur, Figs. 9A2, B2). The ventrolateral otic ridge and the subotic fossa of *Kawichthys* have respectively the same topographic position as the “Querkiel” of *Stethacanthulus* [22] and its ventral fossa. The subotic fossa may have housed the lateral head vein. The ventrolateral otic ridge resembles the ridge situated below the lateral otic fossa in *Akmonistion* [24] and *Orthacanthus* [10], but not labelled by the authors.

The lateral otic ridge does not form a lateral otic process *sensu* Schaeffer [10], contrary to some Paleozoic chondrichthyans (e.g. *Orthacanthus*, *Tamiobatis*). However, a process is present ventral to the ventrolateral otic ridge (vop, Figs. 3A2, B2). It is confluent with the latter immediately posterior to the foramen for the glossopharyngeus nerve (fIX, Fig. 3A2). Then, the process extends posteroventrally. No feature of the endocranial cavity is overlain by this process. This process may represent an articular process for the hyomandibular, which may have been followed further posteriorly by the first branchial arch, innervated by the glossopharyngeus nerve. There is no evidence of a periotic process, unlike in “*Cobelodus*”.

The occipital region is poorly preserved in both specimens. A pair of sharp ventral paroccipital processes is present ventrally (vppr, Figs. 3A2, B2; 6A2, B2; 11A2, B2). They may have flanked ventrally a notochordal canal.

Between the ventral paroccipital processes, the basicranium in both specimens is poorly calcified. This area extends anteriorly and is followed by two laterally divergent grooves. The unpaired, median poorly calcified zone forms thereby a groove for the dorsal aorta (gda, Figs. 6A2, B2). In the two specimens studied here, only the posteroventral part of the braincase is preserved, whereas its posterodorsal part is lacking immediately behind the loop of the posterior semicircular canal, so there is no evidence of an occipital arch in either specimen. In “*Cobelodus*”, a relatively small occipital arch arises immediately posterior to the posterior semicircular canal and is separated from the latter by the dorsal part of the otico-occipital fissure [2]. In “*Cobelodus*”, a small median occipital crest is also situated in the same position as in the specimens studied here. Consequently, it is possible that *Kawichthys* possessed a dorsal otico-occipital fissure, which separated a small occipital arch from the rest of the braincase (oof?, Figs. 3A2, B2; 5A2, B2). The ventral part of the fissure seems to have been a fragile area of the braincase, and it is possible that the latter was broken at this level during fossilization, and that the occipital arch was consequently not preserved. If a “*Cobelodus*”-like otico-occipital fissure was originally present in the specimens studied here, a posterior tectum probably separated it from the endolymphatic fossa (pt, Fig. 5A2). A similar posterior tectum bordering the endolymphatic fossa posteriorly, is also present in *Tamiobatis* [10], *Akmonistion* [24] and *Cladodoides* [1].

#### General features of the endocranial cavity

The endocranial cavity is only partially preserved in both specimens. Only a portion of the posterior part of the endocranial cavity (at the level of the vestibular chamber) and a small part of the anterior part of the endocranial cavity are present in KUVP 56340 (endc, ur, sac, Fig. 9A2). At the level of the parachordal plate, KUVP 152144 displays part of the floor of the posterior part of the endocranial cavity, from the level of the ventral otic process to the orbital opening of the orbital artery. It also shows a portion of the floor of the anterior part of the endocranial cavity (endc, Figs. 8B2, 9B2), and it displays a dorsal dome-shaped endocranial cavity at the level of the orbits. The lateral walls of the dome flare lateroventrally as far as a point where they slope medially (endc, Figs. 3B2, 4A2), and which was probably located at the level of the groove which marks the medial margin of the supraorbital shelf present in KUVP 56340. There is no evidence of the endocranial cavity in the ventral part of the orbital region (see below) and there no evidence of a dorsum sellae (typically located at the level of the bucco-hypophyseal duct).

The canals for the lateral dorsal aortae run forward inside the floor of the braincase (clda, Fig. 8A2). They divide laterally into small, laterally oriented canals for the orbital artery, which reach the lateral wall of the braincase in front of the postorbital process (cora, Fig. 8A2). Anterior to this bifurcation, the canals continue slightly forward (cica, Fig. 8A2), then they are not preserved.

The floor of the posterior part of the endocranial cavity elevates upwards anteriorly (endc, Fig. 9B2). It displays a median groove (nc, Figs. 8B2; 9B2), which merges posteriorly with the poorly calcified part of the basal plate (the groove for the dorsal aortae, see above). The notochordal groove is not roofed. This is apparently not a default of preservation, since the lateral border of the canal merges perfectly with the adjacent floor of the endocranial cavity. This condition is thus similar to that in extant chimaeroids, where the notochord lies free above the basal plate. The notochord is enclosed by cartilage (perhaps derived from the parachordals) in placoderms, primitive actinopterygians, and primitive chondrichthyans.

A precerebral fontanelle may be present, since the anterior end of the dorsal part of the endocranial cavity seems to display a small opening (prcf?; Figs. 5B2).

#### General features of the skeletal labyrinth

**Figure 12:**
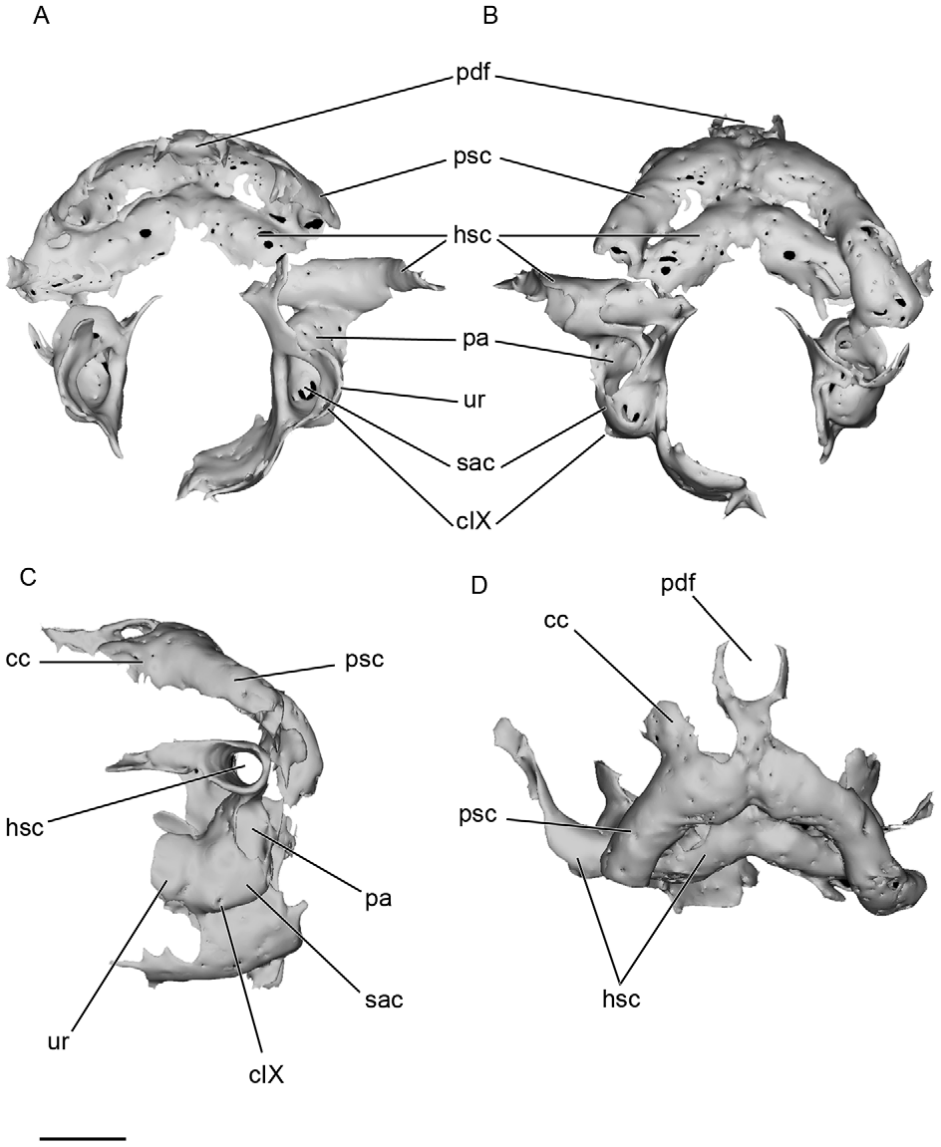
Surface rendering generated from Synchrotron Radiation microtomographic slices of posterior part of the preserved endocranial cavity and skeletal labyrinth of *Kawichthys* KUVP 56340. A, anterior view. B, posterior view. C, right lateral view. D, dorsal view. Scale bar = 0.5 cm. Abbreviations: cc, crus commune; pa, posterior ampulla. See Figs. 5 and 9 for other abbreviation.

The skeletal labyrinth is seen in reconstructions of the endocast. It is only partially preserved in both specimens. The vestibular apparatus appears similar to that of modern chimaeroids, osteichthyans and some Paleozoic chondrichthyans, such as “*Cobelodus*” [2], *Cladodoides* [1] and the Sibyrhynchidae [6] in that the anterior and posterior semicircular canals meet dorsally at a crus commune (psc, cc, Figs. 12C, D).

The vestibular chamber is almost entirely confluent with the cranial cavity (sac, ur, Figs. 9A2, B2; 12A). The medial capsular wall is unchondrified. There is no evidence of a prominent lagenar chamber in the saccular chamber (sac, Figs. 12B, C). The utricular and saccular chambers are relatively small and they meet medially (ur, sac, Fig. 12A). The utricular recess is represented by a low bulge, which is constricted at the level of its junction to the ventral saccular chamber. The posterior ampulla is confluent with the saccular chamber (pa, sac, Fig. 12A–C), and there is no evidence of a preampullary canal. The canal, which leads to the glossopharyngeal foramen, is very short, almost invisible, and its internal opening is situated on the anteroventral part of the saccular chamber (cIX, sac, Fig. 12C).

### Phylogenetic analysis

Cladistic analysis was performed by using a matrix comprising 35 neurocranial characters and 19 taxa in which the neurocranium is quite well preserved, including 15 fossil chondrichthyans (*Akmonistion* [24], *Cladodoides* [1], *Cladoselache* [2], “*Cobelodus*” [2], *Debeerius* [13], *Doliodus* [8], *Egertonodus* [38], *Iniopera* sp. [6], *Kawichthys*, *Helodus* [39], [40], *Orthacanthus* [1], [10], *Pucapampella* [14], [41], *Synechodus* [42], *Tristychius* [34], *Tamiobatis* sp. [10], 3 extant chondrichthyans (*Chimaera* [37], [43], [44], *Notorynchus* [45], *Squalus* [36], [46], [47]), and one osteichthyan (*Mimia* [48]) as single outgroup. Because the aim of the present phylogenetic analysis is not to test the monophyly of the ingroup (i.e., the chondrichthyans), but to determine the interrelationships of some well preserved early fossil taxa within chondrichthyans, we use here a single outgroup. Characters and character states are presented in Text S1. Characters by taxon are presented in Text S2.

**Figure 13:**
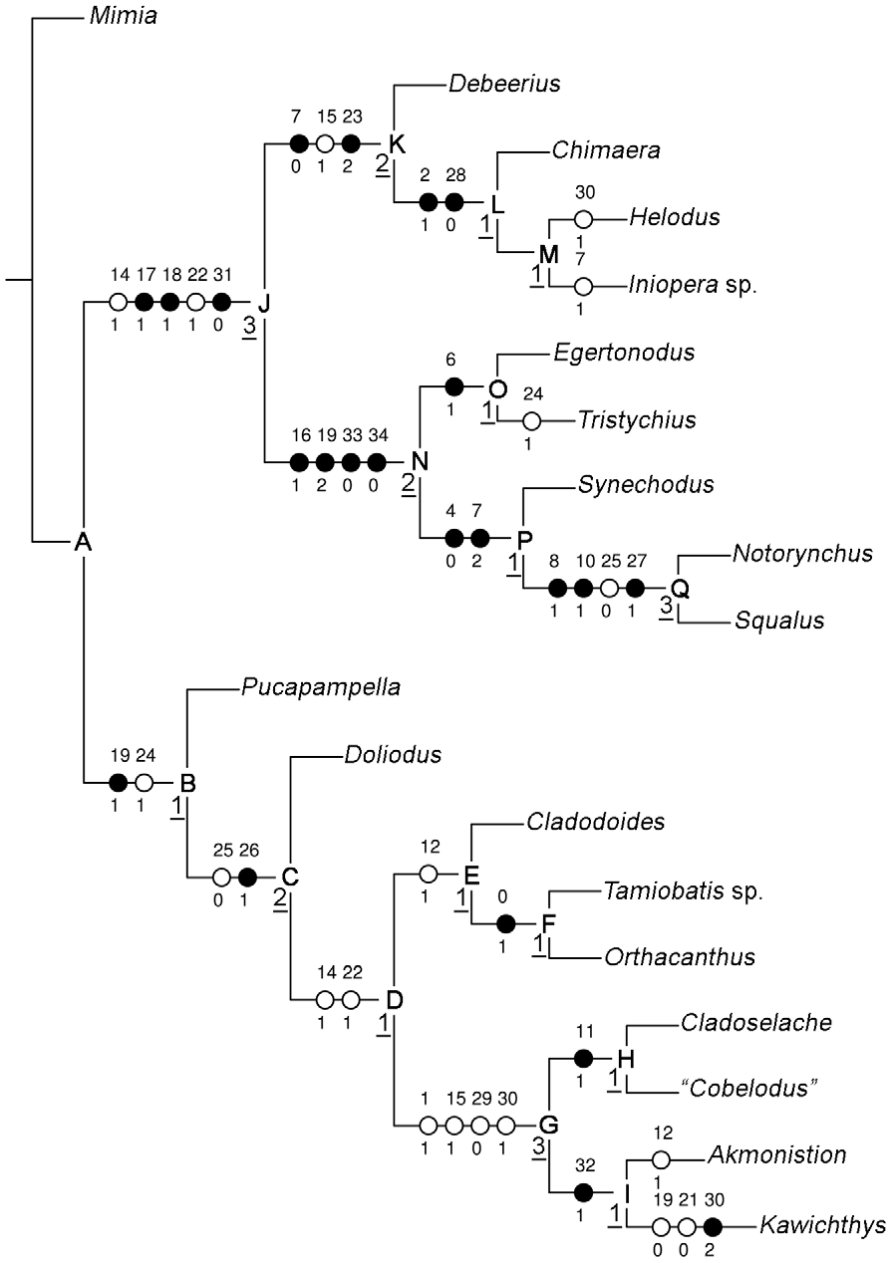
Phylogenetic relationships recovered by a cladistic analysis of 35 neurocranial characters. Length = 65, CI = 0.631, RI = 0.795. White plots, homoplastic synapomorphies; Black plots, non-homoplastic synapomorphies. The ambiguous synapomorphies are not shown. Nodes are indicated by letters. Bremer supports are indicated by an underlined number on the left of the corresponding node.

The heuristic search results in one parsimonious tree (consistency index (CI) = 0.631, retention index (RI) = 0.795), with a total length of 65 steps (Fig. 13).

Node A defines a clade that contains two monophyletic sister groups (node B and J). Node B defines a clade that includes the stem-group chondrichthyans, i.e., *Pucapampella*, which is resolved as a sister group of *Doliodus* plus a large clade that comprises the “cladodont sharks” plus *Orthacanthus* (Node C). Node B is supported by a the presence of a glossopharyngeal nerve floored by the hypotic lamina and exiting through the open metotic fissure (character 19) and a postorbital articulation on the inferred chondrified lateral commissure (character 24). The absence of an ethmoidal articulation (character 25) and an orbital articulation anterior to the optic foramen (character 26) are respectively homoplastic and non-homoplastic synapomorphies of the clade which gathers *Doliodus* and the “cladodont sharks” plus *Orthacanthus* (Node C). The “cladodont sharks” plus *Orthacanthus* (Node D) comprise two monophyletic sister groups: *Orthacanthu*s plus the “ctenacanths” *Cladodoides* and *Tamiobatis* (Node E) on the one hand, and the Symmoriiformes on the other (Node G). The homoplastic synapomorphies at Node D are the presence of a posterior dorsal fontanelle, well-defined and forming an endolymphatic fossa, separated from the otico-occipital fissure by a posterior tectum when the fissure persists in the adult (character 14), and an occipital block which is wedged between otic capsule (character 22). Node E, which unites *Orthacanthus* with the “ctenacanths” (non-monophyletic here), is supported by the presence of dorsal otic ridges forming horizontal crests (character 12). The Symmoriiformes (Node G) are united by the following homoplastic synapomorphies: a tropibasic skull (character 1); a small, rounded and unfloored endolymphatic fossa (character 15); a dorsal aorta which divides into lateral dorsal aortae anterior to the occipital region (character 29); and a dorsal aorta canal (character 30). *Kawichthys* falls within this clade, and it is sister to *Akmonistion* (Node I). This clade is supported by character 32 (distinct foramina for hyoidean artery).

Node J characterises a crown-chondrichthyan clade which contains two monophyletic sister groups: stem and crown-holocephalans on the one hand (Euchondrocephali) (Node K), and hybodontiforms plus neoselachians (Euselachii) on the other (Node N). The crown-chondrichthyan clade (Node J) is well supported by three non-homoplastic and two homoplastic synapomorphies: a posterior dorsal fontanelle, well-defined and forming an endolymphatic fossa, separated from the otico-occipital fissure by a posterior tectum when the fissure persists in the adult (character 14); an occipital block which is wedged between otic capsule (character 22); a closed dorsal part of the otico-occipital fissure in the adult (character 17); a closed metotic fissure in the adult (character 18); and the absence of canals for the lateral dorsal aortae (character 31). The euchondrocephalans (Node K) are united by: a postorbital arcade which is dorsoventrally continuous but does not enclose the jugular vein (character 7); a small, rounded and not floored endolymphatic fossa (character 15); and the absence of a hyomandibular articulation on the neurocranium (character 23). A palatoquadrate that is fused to the neurocranium (character 2) and internal carotid arteries which do not supply the blood to the brain (character 28) are two non-homoplastic synapomorphies of the crown-holocephalans (Node L). Node M is supported by an ambiguous synapomorphy (character 21) by using a fast optimization. The following characters support the euselachians (Node N): a glossopharyngeal nerve which is floored by the hypotic lamina and is enclosed by a glossopharyngeal canal (character 19); the presence of perilymphatic fenestrae (character 16); a chondrified medial capsular wall (character 33); and the absence of a crus commune between the anterior and posterior semicircular canals (character 34).

## Discussion

### Notes on comparative anatomy

#### Probable extent of embryonic cartilages and palatoquadrate articulation in *Kawichthys*

A postorbital articulation on the lateral commissure is present in *Kawichthys* (see above), as in other Symmoriiformes, as well as in *Pucapampella*, xenacanths, “ctenacanths” and *Tristychius*. This postorbital articulation is different from that of Hexanchiformes in which there is no chondrified lateral commissure, hence no continuous postorbital process. In these taxa, the articular facet is located on the primary postorbital process (*sensu* Holmgren [46]), lateral to the otic lateral line nerve and dorsal to the jugular canal (e.g., *Notorynchus* [45]).

The postorbital articulation of *Kawichthys* may have been completed by an anterior orbital articulation between the palatoquadrate and the basitrabecular processes, as demonstrated below.

The posterior margin of the embryonic polar cartilages is commonly marked by the foramen for the pituitary vein in gnathostomes. In the specimens studied here, there is no evidence for any foramen for the pituitary vein. Nevertheless, the bucco-hypophyseal opening represents an important landmark, since it reflects the original position of the bucco-hypophyseal fenestra, which is present in the basicranium at the level of the junction between the embryonic parachordal and polar cartilages in chondrichthyans [36]. The bucco-hypophyseal opening of *Kawichthys* appears located far anterior to the postorbital wall (bho, pwo, Figs. 6A2, B2), while it is usually situated immediately anterior to it in most other chondrichthyans. However, it approximately coincides with the posterior end of the suborbital shelf (subs, Figs. 3A2, B2). Consequently, the inferred embryonic parachordal plate of the specimens studied here extends far anteriorly and forms more than half the basicranium, as in *Doliodus* [8]. The embryonic polar cartilages are therefore inferred to have been located anterior to the bucco-hypophyseal opening, and thereby probably participated in a major part of the suborbital shelf, as in *Cladodoides* [1] and the symmoriiforms [2].

Maisey [1] defined the orbital articulation as an articulation situated immediately anterior to or within the foramen for the efferent pseudobranchial artery and the inferred anterior margin of the polar cartilage-derived region, which forms the basitrabecular process. This articulation either occurs anterior (e.g., *Orthacanthus*, *Tamiobatis*, *Akmonistion*) or posterior to the optic foramen (e.g., *Chlamydoselachus*, *Squalus*, “*Cobelodus*”). In *Kawichthys*, there is no evidence of a foramen or notch for the efferent pseudobranchial artery to indicate the embryonic boundary between the trabecular plate and the polar cartilage. Nevertheless, as mentioned above, the polar cartilages were probably situated far anteriorly and the interorbital plate flares laterally in its anterior part. This suggests that the enlarged part of the interorbital plate may represent a laterally extended basitrabecular process that may have articulated with the palatoquadrate, thus representing an anterior orbital articular surface (orart, Figs. 6A2, B2). If that interpretation is correct, the posterior limit of the embryonic ethmoid plate approximately corresponds to the anterior enlarged part of the interorbital plate; the efferent pseudobranchial artery may have passed adjacent to the latter, as in *Falcatus falcatus* [2], [32]. Consequently, the inferred embryonic trabecular plate formed only the anterior third of the basicranium. A similar condition is also inferred in some Paleozoic chondrichthyans, such as *Cladodoides* [1], the Symmoriiformes [2], and *Doliodus* [8], but differs from that of iniopterygians [6], extant chimaeroids and neoselachians.

#### Tropibasic skull anatomy

In the orbital region of *Kawichthys* the basicranium is very narrow, suggesting both closely-set embryonic polar cartilages and trabeculae, as in most gnathostome tropibasic skulls [2]. The dorsal surface of the interorbital plate is flat and continuous with the ventral part (into, Figs. 7A2; 8B2), unlike the arrangement where the endocranial cavity is present at this level (e.g., *Iniopera* sp. [6]). Consequently, the endocranial cavity in *Kawichthys* probably did not extend ventrally in the mid orbital region. Conversely, a dome-shaped endocranial cavity, in which the lateral walls seem to meet ventromedially, is present dorsally in the orbital region. Anterior and posterior to the orbit, the outer layer of the basicranium is clearly distinct from the inner layer, even though they are very close (ol, il, Figs. 7A1, B). In addition, the shape of the inner wall of the braincase is concave at these levels (endc, Figs. 9B2), and the chordal part of the endocranial cavity seems to elevate upwards anteriorly (endc, Figs. 9B2).

This situation may be interpreted as follows: the endocranial cavity of *Kawichthys* did not extend ventrally in the mid region of the orbits. The eyes were probably separated by an interorbital septum. The latter may correspond to the medially fused orbital cartilages, which were connected ventrally only by membranous or connective tissues to the interorbital plate, and which may have separated the interorbital plate from the dorsally situated endocranial cavity. The chordal part of the endocranial cavity is elevated anterodorsally to join the dorsal dome-shaped endocranial cavity in the orbital region above the interorbital septum.

A tropibasic skull is obviously present in “*Cobelodus*” [2]. Some other members of the Symmoriiformes are suspected to possess a tropibasic skull because an interorbital septum was probably present (*Cobelodus aculeatus* [49] and *Stethacanthulus meccaensis* [2], [22]). A tropibasic skull may be a synapomorphy of the Symmoriiformes. Although the chondrocranium has been restored as platybasic in *Akmonistion* [24], it is dorsoventrally crushed in all the available material and could have been tropibasic originally.

The only apparent difference in the tropibasic condition of “*Cobelodus*” and the specimens studied here is the inferred extent of the embryonic polar cartilages, which apparently extended farther anteriorly in *Kawichthys* than in “*Cobelodus*”. The *Kawichthys* condition may be inferred in some other symmoriiforms, such as *Stethacanthulus* and *Akmonistion*, since these also show a narrow suborbital shelf which extends farther anteriorly than in “*Cobelodus*”. Nevertheless, even if the inferred embryonic polar cartilages of “*Cobelodus*” are less extensive than in the specimens studied here, they also appear hypertrophied, compared to Recent elasmoranchs [2]. In “*Cobelodus*”, the anterior part of the interorbital septum (i.e. the inferred embryonic preoptic pila) is not chondrified, nor calcified when it meets the inferred embryonic trabeculae, which are more extensive backwardly towards the orbit than in the specimens studied here. In *Stethacanthulus*, the preoptic pila is apparently better developed, hence the chondrified anterior part of the interorbital septum. This may be correlated to the anterior extent of the inferred embryonic polar cartilage, if its neurocranium is compared to that of the specimens studied here.

To summarize, a tropibasic skull is widespread in the Symmoriiformes, but two slightly different conditions can be recognized. The hypertrophy of the polar cartilage and the deepening and expansion of the orbit are probably correlated to the formation of an interorbital septum and a tropibasic skull. Nevertheless, the polar cartilages of *Kawichthys* (and probably *Akmonistion* and *Stethacanthulus*) were probably more extensive anteriorly than those of “*Cobelodus*”. This may be correlated with the different conditions of the anterior part of the interorbital septum in these taxa.

#### Glossopharyngeal nerve and hypotic lamina

In modern elasmobranchs, the metotic fissure, i.e. the ventrolateral continuation of the otico-occipital fissure, forms an embryonic space between the floor of the otic capsule and the hypotic lamina, which is the lateral extension of the parachordal plate [36]. The ontogenetic obliteration of the metotic fissure by a posterior basicapsular commissure only leaves a canal for the glossopharyngeus nerve, which passes below the otic capsule and opens posteroventrally (e.g., neoselachians and hybodonts). When the fissure remains open in the adult (e.g., *Cladodoides*, “*Cobelodus*”), the glossopharyngeus nerve passes through the fissure between the otic capsule and the hypotic lamina. In taxa where there is no hypotic lamina, such as the holocephalans and osteichthyans, the glossopharyngeus nerve passes through the otic capsule and exits the neurocranium by a short canal (e.g., *Iniopera* sp.), and a neoselachians-like glossopharyngeus canal is absent.

In *Kawichthys*, the course of the glossopharyngeal canal recalls that of holocephalans and osteichthyans. Nevertheless, an otico-occipital fissure may be present. In addition, a posterior basicapsular commissure, which connects the inferred parachordal plate to the otic capsule, may be present immediately posterior to the ventral otic process, posteroventrally to the foramen for the glossopharyngeus nerve (pbc?, 3A2). This condition is unknown among extant and Paleozoic adult sharks. Nevertheless, the ventral part of the otico-occipital region in osteichthyans includes a basicapsular fenestra (or vestibular fontanelle) and the anterior part of the metotic fissure (through which the glossopharyngeus nerve passes). The latter is delimited posteriorly by a posterior basicapsular commissure. This osteichthyan condition may thus be present in the specimens studied here, but with a closed vestibular fontanelle and a mostly closed metotic fissure.

#### Arterial supply to the brain

According to the 3D reconstructions of the braincases and the determination of the different foramina and grooves, the arterial cranial circulation of *Kawichthys* can be reconstructed as follows.

**Figure 14:**
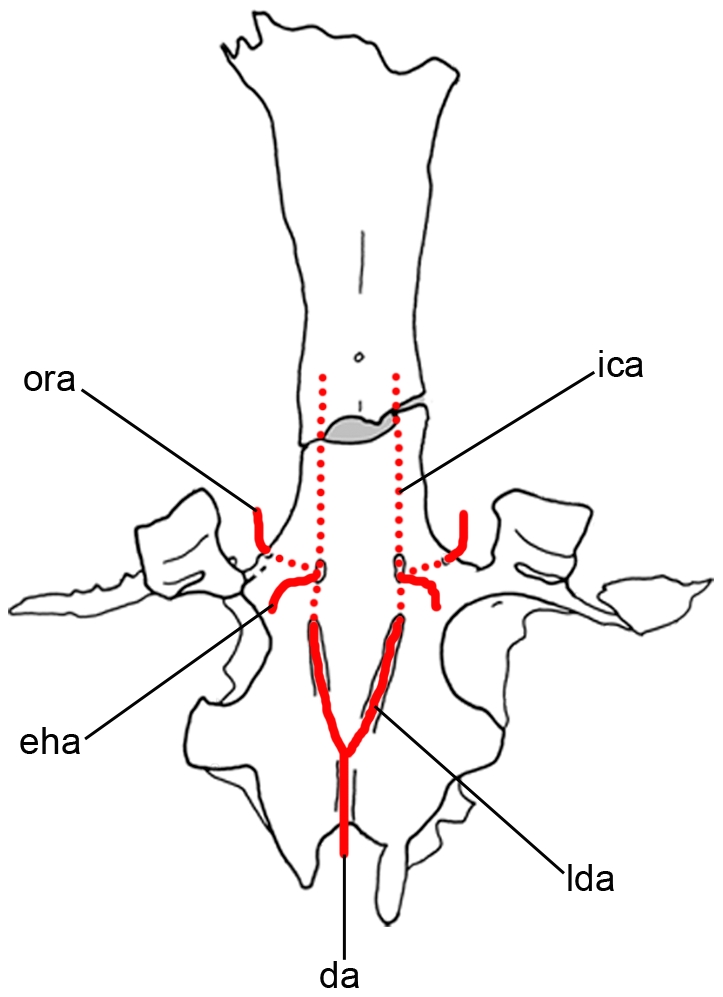
Reconstruction of the basicranial arterial circulation of *Kawichthys* in ventral view. Solid lines represent vessels visible on the surface and dashed lines represent those internal to the cartilage. Not to scale. Abbreviations: da, dorsal aorta; eha, efferent hyoidean artery; ica, internal carotid artery; lda, lateral dorsal aorta; ora, orbital artery.

As in symmoriiforms, the lateral dorsal aortae diverged from the dorsal aorta anterior to occipital level (da, lda, Fig. 14). Contrary to the condition in many Symmoriiformes [2], there is no evidence of a dorsal aortic canal, but the dorsal aorta apparently ran forward into a deep median groove (gda, Figs. 6A2, B2). As in other symmoriiforms, the dorsal aorta gave off two laterally divergent lateral dorsal aortae, which were also housed inside grooves (glda, Figs. 6A2, B2). Then, the lateral dorsal aortae entered the basicranium (flda, Figs. 6A2, B2) via large internal canals (clda, Figs. 8A2). Enclosure of the lateral dorsal aortae within the parachordal cartilage is a widespread feature among Paleozoic chondrichthyans (e.g., *Pucapampella*, *Cladodoides*, *Tamiobatis*, “*Cobelodus*”, *Akmonistion*, *Orthacanthus*, *Doliodus*), whereas the aortic system is free in modern chondrichthyans, as well as in hybodonts, *Acronemus* and the iniopterygian *Iniopera* sp. Each lateral dorsal aorta gave off respectively from back to front, an efferent hyoidean artery (eha, Fig. 14), which exited the basicranium at the level of the postorbital wall (feha, Fig. 6A2), and an orbital artery (ora, Fig. 14), which exited the lateral wall of the braincase immediately anterior to the postorbital wall. *Akmonistion* is assumed to have possessed similarly distinct foramina for the efferent hyoidean arteries [24]. The lateral dorsal aortae of *Kawichthys* did not exit the braincases to pass along the ventral surface of the basicranium and re-enter the braincase through the bucco-hypophyseal opening. Instead, these vessels probably ran forward inside the neurocranium through canals (as the internal carotid arteries; ica, Fig. 14), which seem to follow the chordal part of the endocranial cavity. As previously seen, the latter elevates anterodorsally to probably meet the dorsal dome-shaped endocranial cavity above the interorbital septum. Consequently, the internal carotid arteries probably remained internal, and entered the endocranial cavity dorsal to the interorbital septum. From there, the internal carotid arteries probably divided into optic, ophthalmic and basilar arteries.

The pattern present in *Kawichthys* appears unique among Paleozoic chondrichthyans. In xenacanths, “ctenacanths” and other symmoriiforms, the lateral aortae were enclosed inside canals for most of their length, exiting the braincase only where they divided into the internal carotid, orbital and efferent hyoidean arteries, and the internal carotids entered the braincase either through the hypophyseal fenestra or via separate foramina. However, the internal arrangement of major blood vessels is known only in a few Paleozoic sharks, and their course is inferred in most cases purely on the basis of their different external foramina. In some taxa, the internal carotid arteries were apparently enclosed completely by canals (e.g., the Cretaceous hybodont *Tribodus limae* [3]).

### Notes on the phylogeny

The overall topology of the tree proposed here strongly differs from most of those recently published in which either the xenacanths, “ctenacanths” and symmoriiforms are related to extant neoselachians and/or hybodontiforms [10]–[13], or in which the symmoriiforms emerged as stem-group holocephalans [15]–[17], and in which xenacanths and “ctenacanths” were more closely related to neoselachians and hybodontiforms. Actually, the tree topology found here broadly supports the phylogenetic hypothesis based on tooth morphology presented by Ginter et al. [19]. This phylogenetic hypothesis unites the hybodontiforms and neoselachians plus the stem and crown-holocephalans in a crown-chondrichthyan clade, whereas xenacanths, phoebodonts, “ctenacanths” and symmoriiforms are situated in a stemward position, before the divergence of euselachians and euchondrocephalans. Ginter and Piechota [50] noticed that the general structure of the tooth base in chondrichthyans is conservative, so that it can be a diagnostic feature at a higher systematic level, whereas the tooth crown is a highly adaptive structure. In fact, neoselachians, hybodontiforms, stem-holocephalans and crown-holocephalans share a euselachian type of tooth base morphology, whereas xenacanths, the Phoebodontiformes, “ctenacanths” and the Symmoriiformes possess a different, supposedly more primitive, kind of tooth base morphology. [18], [19]. This crown-group chondrichthyan clade is now supported by neurocranial synapomorphies: a closed dorsal and ventral part of the otico-occipital fissure and a free aortic system below the neurocranium (the condition in *Helodus* may be autapomorphic, since its dorsal aorta canal does not give rise anteriorly to two divergent lateral dorsal aorta canals, and is thus different from that of the Symmoriiformes). Besides these dental and neurocranial characters, some postcranial features may represent additional synapomorphies of crown-chondrichthyans: a mandibular joint between the palatoquadrate and Meckel's cartilage located anteroventral to the rear of the braincase (present in stem and crown-holocephalans, hybodontiforms, and non-hexanchoid neoselachians, whereas it is situated posterior to the occiput in xenacanths, “ctenacanths” and symmoriiforms); a shoulder joint with strong propterygial support, and an enlarged pelvic propterygium. These characters are, for instance, readily observed in *Iniopera* [6], [7], [51].

The “cladodont sharks” are represented in the present work by *Cladodoides*, *Tamiobatis*, *Akmonistion*, *Cobelodus* and *Cladoselache* (the teeth are unknown in *Kawichthys*). “Cladodont” teeth have a large, conical, central cusp, two or more smaller lateral cusps of similar shape, and a flattened, disc-like base [52]. The xenacanth *Orthacanthus* falls into the “cladodont” group in our phylogeny, but possesses diplodont teeth, although the tooth base is of a primitive type. Our phylogeny supports Ginter et al.'s argument [19] that diplodont teeth of xenacanths are a derived condition and that *Doliodus*, which also possesses diplodont teeth, is not a stem xenacanth, contra Mader [53] and Long and Young [54]. However, our phylogeny differs by suggesting that “cladodont sharks” are paraphyletic unless xenacanths are included, rather than a monophyletic extinct sister group to crown-group chondrichthyans. It has been suggested elsewhere that the diplodont teeth of xenacanths are in fact of modified cladodont design, with enhanced lateral cusps and the central cusp slender, small or even absent [55], and Ginter [56] has suggested that xenacanths are derived from the phoebodontiforms. “Cladodont” sharks are further united with xenacanths by the presence of a palatoquadrate with a deep and elongated otic portion articulating with the postorbital process, and a narrow orbital ramus extending anteriorly. Some postcranial characters are also shared by “cladodont sharks”, such as a shoulder joint with strong metapterygial support [57].

Although the diplodont teeth of *Doliodus* are fused into a whorl [19], so far as the cusps are concerned, they seem to be more closely similar to the primitive type than to the euselachian type. This supports the position assigned to this taxon in the present tree topology.

Our tree topology also agrees with the distribution of the different tooth types through time. The earliest known tooth occurrence consists of diplodont teeth (*Leonodus*, *Doliodus*) from the Lower Devonian. The earliest omalodontiform teeth appear in the upper part of the Lower Devonian, while the earliest teeth of primitive and euselachian types, respectively represented by the first cladodonts (and the diplodonts *Antarctilamna* and *Phoebodus*) on the one hand, and *Protacrodus* on the other, appear the fossil record simultaneously during the Givetian (Middle Devonian). There is some possibility that all the cladodont type teeth may have evolved from a diplodont type, while the entire range of hybodonts, neoselachians, stem and crown holocephalan teeth or tooth plates can by derived from a protacrodont model [19].

While the Symmoriiformes are usually considered to be phylogenetically related to other Paleozoic “cladodont” chondrichthyans, a phylogenetic relationship has been proposed between the Euchondrocephali and Symmoriiformes [15]–[17]. Maisey and Lane [31] pointed out a potential neurocranial synapomorphy; a small, rounded and unfloored endolymphatic fossa (character 15); which may support such a relationship. According to the present topologies, however, this character appears convergently in the Euchondrocephali and Symmoriiformes, and thus is not a secondary homology for these taxa. Although the Symmoriiformes and holocephalans display a similar morphology of the endolymphatic fossa, the origin of its posterior boundary seems different; in the Symmoriiformes, the posterior tectum separates the endolymphatic fossa from the otico-occipital fissure. Unlike in neoselachians, the posterior tectum of the Symmoriiformes was probably not derived ontogenetically from the embryonic synotic tectum, nor from the occipital arch, since the floor of the fossa is not chondrified (hence the absence of a synotic tectum posterior to the anterior extremity of the fossa) and the posterior tectum is separated from the occipital arch by a persistent otico-occipital fissure. The posterior tectum in this case thus seems to be derived from a separate embryonic taenia tecti medialis, situated at the posterior end of the fossa [31]. Unfortunately, the ontogeny of the otic region in extant holocephalans is still poorly known. However, there is also no chondrified floor in the fossa, suggesting that the posterior tectum is not derived from the synotic tectum. The otico-occipital fissure is closed during the ontogeny, contrary to the condition in the Symmoriiformes; and the auditory capsule is similar to that of osteichthyans. By comparison, in osteichthyans in which the otico-occipital becomes closed during the ontogeny, the auditory capsule and occipital arch are connected across the otico-occipital fissure, so that the posterior tectum behind the parietal fossa is apparently formed by the occipital arch [36]. Consequently, the posterior tectum of chimaeroids may also be a derivative of the occipital arch, as in some neoselachians and osteichthyans, rather than a separate embryonic taenia tecti medialis; unfortunately, chimaeroid ontogeny is still poorly known, and the origin of of its posterior tectum remains uncertain. Nevertheless, in extant holocephalans (as well as in *Iniopera*), much of the occipital arch extends between the otic capsules, as in neoselachians, whereas, it extends only on a short distance between the rear of the otic capsules in the Symmoriiformes, xenacanths and “ctenacanths”. This suggests that the origin of the posterior tectum of chimaeroids and neosolechians is probably the same (except for some neoselachians in which the posterior tectum is formed from the synotic tectum), while the posterior tectum of the xenacanths, “ctenacanths” and Symmoriiformes probably has a different ontogenetic origin (probably a separate embryonic taenia tecti medialis).

This hypothesis is also supported by the distribution of the character 14 in the present tree topology. The state 1 of this character (posterior dorsal fontanelle well-defined, separated from the otico-occipital fissure by a posterior tectum even when the fissure persists in the adult) appears convergently at Node J (crown-group chondrichthyans) and in Node D (“cladodont sharks” plus *Orthacanthus*), so that this states is actually not a secondary homology in these taxa. This could be explained if the formation of the posterior tectum was different in these taxa. The different morphology of the fossa in crown-group chondrichthyans (floored by cartilage in neoselachians, but not in chimaeroids) is probably due to the specialization toward low-frequency semidirectional sound detection in neoselachians. The different morphology of the posterior dorsal fontanelle of “cladodont sharks” plus *Orthacanthus* (anteroposteriorly elongated) and the Symmoriiformes (slot-shaped) may be explained by the anteroposteriorly elongated otico-occipital region of the former, while this region is comparatively short in the Symmoriiformes.

Janvier [15] and Coates and Sequeira [16], [17] pointed out another possible synapomorphy of a clade gathering the Symmoriiformes and the holocephalans: the presence of small calcified ring- or C-shaped scales that strengthen the lateral-line canals. This kind of scale is indeed only present in chimaeroids among modern chondrichthyans. However, the lateral line is also supported by a series of calcified ring- or C-shaped scales, as in holocephalans and symmoriiforms, in some fossil euselachians: the cretaceous galeomorph *Mesitaea sahelalmae* (which otherwise lacks denticles, like symmoriiforms and holocephalans) [58]; the Upper Jurassic squalomorph *Protospinax annectans* [59]; and possibly the hybodont *Hybodus fraasi* [60]. Ørvig [61] also noticed that free latero-sensory elements, although with a different shape than those of chimaeroids, may be present in a xenacanth figured by Fritsch [62]. Actually, the condition in Paleozoic holocephalans is more diversified than in extant chimaeroids, some taxa possessing ring-shaped scales similar to those of extant chimaeroids (e.g., *Debeerius* [13]), while others possessed modified monocuspid scales (e.g., *Echinochimaera* [63]) or tubular elements enclosing the lateral lines (e.g., *Menaspis* [40], [61]). In *Deltoptychius*, the lateral lines are almost entirely included into the dermal shield; and iniopterygians do not posses lateral-line scales. A latero-sensory component of the dermal skeleton appears to be a widespread feature among early gnathostomes, although the shape of the scales may differ from that of chimaeroids: latero-sensory mineralized elements are also found in some osteichthyan fishes, in arthrodires, in some acanthodians and even in some osteostracans [61]. Considering these observations, Ørvig [61] pointed out that a latero-sensory component of the dermal skeleton, in the form of many separate, small calcified elements lining the lateral line canals, may have appeared early in the history of the gnathostomes. Consequently, the phylogenetic status of this character should be reconsidered.

A recent paper on molecular phylogeny of extant chimaeroids provides a timetree derived from a relaxed molecular clock Bayesian method, which suggests that the holocephalans and elasmobranchs (crown-chondrichthyans) diverged in the Silurian about 420 Ma [64]. Our present data agree with this result, since crown chondrichthyan neurocranial specializations can be traced back to at least the Upper Carboniferous (300 Mya) (e.g., *Iniopera* for euchondrocephalans and *Tristychius* for euselacians). Stem holocephalans can be traced back to at least the Upper Carboniferous, while stem neoselachians can be traced back to either the Late Permian (250 Mya) based on the putative fossil record of Synechodontiformes [65], or the Late Carboniferous if *Cooleyella* is a neoselachian [66]. Euchondrocephalans and euselachians are the only chondrichthyan clades that have survived until today. If the current phylogenetic analysis is confirmed by further investigations, the study of the anatomical synapomorphies of the group gathering euselachians and euchondrocephalans will provide valuable data in order to understand their respective selective advantages. For instance, the closure of the otico-occipital fissure during ontogeny occurs in euselachians and euchondrocephalans. Nevertheless, while this condition is probably linked to the development of the low-frequency semidirectional sound detection in neoselachians [31], there is no functional explanation for it in extant chimaeroids. The sensory system of extant chimaeroids, as well as their ontogeny, is still poorly understood [67]. More paleontological and embryological data on extant chimaeroids are therefore needed, while the previous studies on modern chondrichthyans were focused mainly on sharks. Although the holocephalans display several specializations (e.g., a palatoquadrate fused to the neurocranium) and crown-group chondrichthyan synapomorphies, they also retain many plesiomorphic neurocranial characters that are not present in neoselachians (e.g., the structure of the otic capsule). Moreover, recent molecular studies on the extant holocephan *Callorhinchus milii* suggest it might be a useful model for cartilaginous fish because of it has a relatively small genome (910 Mb) and it retains a large number of ancestral vertebrate genes [68]–[71].

Besides these molecular studies, the description of new three-dimensionally preserved braincases of Paleozoic chondrichthyans and extant holocephalan embryos by means of the computerized X-ray synchrotron microtomography (a powerful tool for non-destructive imaging of fossil and living forms) will provide valuable data for elucidating chondrichthyan phylogeny and gnathostome interrelationships.

## Supporting Information

Text S1 **List of Characters and character states used for the phylogenetic analysis.**
(DOC)

Text S2 **Data Matrix used for the phylogenetic analysis.**
(DOC)
