# Supplementary material for: A New Paleozoic Symmoriiformes (Chondrichthyes) from the Late Carboniferous of Kansas (USA) and Cladistic Analysis of Early Chondrichthyans
Source: PLoS One. 2011 Sep 27;6(9):e24938. doi: 10.1371/journal.pone.0024938 (PMC3181253; doi:10.1371/journal.pone.0024938)
Supplement: Text S1 — List of Characters and character states used for the phylogenetic analysis. (DOC) [file pone.0024938.s001.doc]

**Text S1 – List of Characters and character states used for the phylogenetic analysis**

The numbers refer to the characters, which are followed by the references to previous uses and their detailed definition. The data matrix is given in Text S2.

0. Multilayered prismatic calcification in the outer wall of the neurocranium absent (0), or present (1). [1, 2].

A single layer of hydroxyapatite prisms lines the inner and outer wall of the neurocranium in Recent and most extinct chondrichthyans. Nevertheless, some Paleozoic chondrichthyans display multilayered prismatic calcification in the outer wall of the neurocranium (e.g., *Orthacanthus*, *Tamiobatis* [3]). To date, the significance of multiple prismatic layers is still under discussion.

In *Egertonodus basanus*, the neurocranium is mostly composed by one layer of prisms: there are apparently two or three prism layers only around the posterior semicircular canals, but not in all part of the outer wall of the neurocranium [4]. This condition is thus different from the multilayered calcification of *Orthacanthus* and *Tamiobatis*.

1. Tropibasic skull absent (0), or present (1). [5].

Following Maisey [6], here we use the terms “platybasic” and “tropibasic” only with reference to the morphology of adult extant and fossil chondrichthyans. These terms refer to the general topology of the basicranium. An embryological definition (i.e. platytrabic and tropibasic, see [6] for discussion) is practically untestable for fossils. A tropibasic skull displays an interorbital septum and the cranial cavity separates the orbits only dorsally and posteriorly. By contrast, a platybasic skull is defined by the lack of an interorbital septum in the floor of the cranial cavity and by the separation of the ventral side walls of the left and right orbits by the cranial cavity.

The skull of holocephalans displays an interorbital septum. Nevertheless, this septum is formed from the orbital cartilage and not by a trabecula communis and is situated dorsal to the brain and cranial cavity. The skull is thus platybasic. In *Helodus*, Moy-thomas [7] described an interorbital septum containing a large fenestra though which the orbits were probably in contact. Nevertheless, the author states “it is not possible to be sure whether this space really lay above the brain” (p. 495). Patterson [8] re-examined the *Helodus* material and “the best preserved neurocranium seems to show a rather wide cranial cavity between the eyes”. Nevertheless, both these interpretations infer a platybasic pattern.

*Akmonistion* and *Cladoselache* share with “*Cobelodus*” and other symmoriiforms many features, such as very large orbits [6]. Nevertheless, it is not possible to determine whether an interorbital septum like that of “*Cobelodus*” is present, even though other symmoriiforms clearly display this feature (e.g. *Stethacanthulus meccaensis*; [6, 9]). A tropibasic skull is considered to be present in *Kawichthys*.

2. Palatoquadrate fused to the neurocranium absent (0), or present (1). [2].

A holotylic jaw suspension (i.e., the palatoquadrate is fused to the neurocranium) is a classical chimaeroid synapomorphy. According to Lund and Grogan [10], this condition is correlated with the important lateral development of the basitrabecular process, which is a polar cartilage derivative [11]. A fused palatoquadrate is also present in *Helodus* and the Sibyrhynchidae among Paleozoic taxa included in the analysis.

3. Precerebral fontanelle in adults absent (0), or present (1). [2, 3, 5, 12, 13].

The presence of a precerebral fontanelle in adults has been regarded as an elasmobranch synapomorphy [14], but some authors have suggested that the ethmoid canal of holocephalans, which encloses the profundus ramus of the trigeminal nerve and the superficial ophthalmic complex, is homologous to the precerebral fontanelle [3]. Following the discussion about this character provided in [15], here is considered that the precerebral fontanelle of elasmobranchs and the ethmoid canal of holocephalans are not primary homologues.

4. Internasal plate separating the two palatoquadrates absent (0), or present (1).

Neoselachians display both a continuous dental arcade and a palatoquadrate symphysis, which may be well developed between the two palatoquadrates (e.g., *Squalus*) or more flexible (e.g., *Chlamydoselachus*). The same condition occurs in *Synechodus* [16]. This arrangement has been considered as a primitive feature of crown-group gnathostomes [17]. However, the palatoquadrates of extant chimaeroids, although fused to the braincase in the adult, are separated, and the internasal plate supports “vomerine” tooth plates [18], forming with the tooth plate of the palatoquadrate a continuous dental arcade. The same condition is present in iniopterygians and *Helodus* [7, 15]. In addition, in many Paleozoic chondrichthyans (i.e. *Egertonodus* [4], *Cladodoides* [1], *Doliodus* [19], *Pucapampella* [20], xenacanths [3], *Tristychius* [21], *Akmonistion* [13], *Debeerius* [22]), as well as in osteichthyans [23], placoderms [24] and *Acanthodes* [25], there is no palatoquadrate symphysis, and the two palatoquadrates are separated by an internasal plate.

5. Ectethmoid process absent (0), or present (1). [3, 13].

An ectethmoid process is a ventral process which arises from the posteroventral margin of the postnasal wall. It is formed from a ventral and recurved extension of the embryonic lamina orbitonasalis in some modern elasmobranchs, such as hexanchiforms and squaliforms [3, 26]. Among the fossil chondrichthyans used for the present analysis, it is assumed to be present in *Egertonodus* [4] and *Tristychius* [4, 13, 21].

6. Foramen for the trochlear nerve situated anterior to the optic foramen (0), or posterior (1). [5, 12, 13].

Among the taxa studied in the present work, only *Egertonodus* [4] and *Tristychius* [13, 21] show the foramen for the trochlear nerve anterior to the optic one. Schaeffer [3] first assumed that the foramen for the trochlear nerve is anterior to the optic one in *Orthacanthus*, but Maisey [4] revisited this question on the basis of better preserved material, and could show that the foramen for the trochlear nerve is in fact situated posterior to the optic foramen.

7. Postorbital arcade dorsoventrally continuous but does not enclose the jugular vein (0), flares laterally and encloses the jugular vein (1), or reduced to the dorsal part and does not enclose the jugular vein (2).

In gnathostome fossils, a laterally expended postorbital arcade which encloses the jugular vein is considered to be homologous to the chondrified lateral commissure of some Recent neoselachians and osteichthyans [1, 23, 27]. Among the chondrichthyans studied here, such a condition (state 1 of the present character) is present in *Akmonistion* [13], *Egertonodus* [4], *Pucapampella* [20], *Cladodoides* [1], “*Cobelodus*”, *Cladoselache* [6], *Tristychius* [28], *Doliodus* [19], *Orthacanthus*, *Tamiobatis* [3] and *Kawichthys*.

In *Squalus*, the lateral commissure is not fully chondrified, and it is absent in *Notorynchus*, and a continuous, laterally expended postorbital arcade is also absent. Consequently, the postorbital process is only formed from the dorsal primary postorbital process [27]. In fossils in which the postorbital arcade does not flare laterally to enclose the jugular vein, so that the postorbital arcade is reduced to its dorsal part, as in *Synechodus*, we cannot know whether it is because of an absence of lateral commissure, or the unchondrification of this feature. Consequently, no distinction is made between the condition present in *Squalus*, *Notorynchus* and *Synechodus* in the present work (state 2 of the present character).

In extant chimaeroids, there is apparently no lateral commissure, since this feature is correlated with the presence of a spiracular canal in the adult neoselachians [29]. Yet a spiracle is present in the chimaeroids embryos, and some sharks possess a spiracular canal but not a lateral commissure (e.g., *Notorynchus* [27]). In Recent chimaeroids, the jugular vein passes dorsoventrally through the posteromedial part of the suborbital shelf via a cranioquadrate passage, which is formed from the junction of the basitrabecular process, parachordal and hypochordal cartilages, and the periorbital otic process [30, 31]. The lateral wall of the jugular canal is therefore formed from the periorbital process, which is apparently a ventrolateral extension of the otic capsule. The postorbital arcade meets ventrally the periorbital process and thus does not enclose the jugular vein (state 0 of the present character). Unfortunately, we are constrained by the available data on the chimaeroid ontogeny to compare the morphology of the postorbital arcade and suborbital shelf of the chimaeroids to those of neoselachians. Without any further embryological data, it is difficult to know whether the embryonic origin of the periorbital process is actually from a lateral commissure, or not. Nevertheless, the jugular vein keeps on a more ventral than that of neoselachians and Paleozoic shark which possess a chondrified lateral commissure. Consequently, we consider here that these observations lead to create two different states of the character, without any reference to the lateral commissure.

Similar condition as that of extant holocephalans is present in *Helodus* [7] and *Debeerius* [22].

In *Iniopera*, the jugular canal pierces a laterally expended postorbital arcade, above the vagus, glossopharyngeus and hyomandibular nerve, as does in neoselachians and Paleozoic chondrichthyans which possess a chondrified lateral commissure. Consequently, we consider here that *Iniopera* display the same condition for the present character as, for instance, Symmoriiformes, xenacanths and ctenacanths (state 1). Nevertheless, the inferred embryonic origin of the postorbital arcade may be either from a lateral commissure, or from a dorsally situated periorbital process (if these two features are not homologues), since the otic capsules are situated in a extreme dorsal position in this taxon and a cranioquadrate passage was also probably present, in a more dorsal position than that of Recent chimaeroids [15].

8. Embryonic basal angle retained in the adult absent (0), or present (1). [3].

During the embryonic development of orbitostylic neoselachians, the polar cartilage leans against the anterior part of the parachordal cartilage to form an almost 45° angle, even after the trabeculae move to a position almost parallel with the parachordal plate [11, 27]. This so-called basal angle is formed in the polar cartilage, and it involves the palatobasal (orbital) articulation with the palatoquadrate. It is retained in adults chondrichthyans only in some hexanchiforms and squaliforms, such as *Squalus* and *Notorynchus*. The fossils included in the present analysis do not possess a basal angle.

9. Lateral otic process absent (0), or present (1). [2, 3, 5, 12, 13].

Several Paleozoic chondricthyans have a pair of lateral otic processes, which represent an extension of the posterolateral capsular wall, and bear laterally part of the hyomandibular articulation (*Orthacanthus*, *Tamiobatis*). Their lateral otic processes contain the loop of the posterior semicircular canal, and Schaeffer [3] specified that they provide extensive insertion areas for the epaxial musculature by forming, with the dorsal otic ridge, a dorsolateral otic fossa. In *Cladodoides*, a similar process is probably present [1], but its original extent is unknown. Coates and Sequeira [13] considered that such a lateral otic process is present in the symmoriiform *Akmonistion*. Nevertheless, Maisey [6] and Maisey and Lane [32] challenged this interpretation, so that the condition is considered as doubtful for this taxon in the present analysis.

10. Postotic process absent (0), or present (1).

In neoselachians, there is another kind of otic process, the postotic process [1, 27, 32], which enclosed the glossopharyngeus nerve but not the posterior semicircular canal, and lies dorsal to the hyomandibular fossa.

11. Periotic process absent (0), or present (1).

In “*Cobelodus*” [6], there are no otic processes that enclose the loop of the posterior semicircular canal, but a periotic process (*sensu* [6]) located on the lateral capsular wall overlies the horizontal semicircular canal. There is no evidence of a hyomandibular fossa in “*Cobelodus*”, and the hyomandibula was probably attached to the periotic process. Maisey [6] mentioned that there is no lateral otic process in *Cladoselache*, but a periotic process is probably present in at least one or two specimens (J. Maisey, pers. com.).

12. Dorsal otic ridge forming a horizontal crest and flanking the endolymphatic fossa anteroposteriorly absent (0), or present (1).

The anterior semicircular canal may be overlain by a dorsal otic ridge, which flanks the endolymphatic fossa posteriorly. Theses ridges are commonly present in Paleozoic chondrichthyans, but are more developed in some taxa than in other. In *Orthacanthus*, *Tamiobatis* and *Akmonistion*, the posteriormost extremities of these ridges terminate as a horizontal crest. The most pronounced part of the dorsal otic ridges is roughly parallel to the anteroposterior axis.

13. Postorbital arcade anterior to the otic capsule (0), or lateral (1).

In hybodonts, *Tristychius*, *Synechodus*, neoselachians, *Iniopera* and *Kawichthys*, the postorbital arcade or process is situated lateral or immediately posterior to the anterior ampulla.

In the present analysis, the other chondrichthyans in which the inner ear is known display a postorbital arcade positioned entirely anterior to the otic capsule.

14. Posterior dorsal fontanelle connected to the persistent otico-occipital fissure (0), or well-defined, separated from the otico-occipital fissure by a posterior tectum even when the fissure persists in adult (1).

According to Maisey and Anderson [20] and Maisey [33], there is some developmental and paleontological evidence that the endolymphatic fossa of modern elasmobranchs is actually homologous with the posterior dorsal fontanelle of osteichthyans, albeit in a highly modified condition. Indeed, in *Pucapampella* [2] and *Doliodus* [19], the endolymphatic fossa is continuous with the otico-occipital fissure, so that this dorsal opening recalls the posterior dorsal fontanelle of early osteichthyans [23]. It is possible that the endolymphatic fossa was primitively part of the otico-occipital fissure. In modern neoselachian embryos, the closure of the endolymphatic fossa posteriorly is brought either by the fusion of the occipital pilae to the otic capsules (e.g. *Squalus*), or by a posterior extension of the synotic tectum (e.g., *Heterodontus*) [1, 27, 32]. The occiput is wedged between the otic capsules, and there is no persistent otico-occipital fissure, so that the posterior tectum in the adult may be reduced or absent. In many Paleozoic sharks, such as the Symmoriiformes, xenacanths and “ctenacanths”, a posterior tectum separates the endolymphatic fossa from the otico-occipital fissure. In extant chimaeroids, there is no posterior dorsal fontanelle similar to that of neoselachians. Instead, the paired endolymphatic ducts reach directly the surface of the neurocranium, even though they meet immediately before it [3]. Nevertheless, considering that the unpaired “endolymphatic duct” of chimaeroids is located in the same position as the endolymphatic fossa of elasmobranchs, and both house the paired endolymphatic canals and their associated sacs [30], we consider that both structures are homologous with the posterior dorsal fontanelle of osteichthyans.

In the different reconstructions of *Helodus*, there is no evidence of such a posterodorsal opening [7, 8], but it could be the consequence of a poor preservation. Therefore its condition in this taxon is considered as uncertain.

15. Small, rounded and unfloored endolymphatic fossa absent (0), or present (1). [2, 5].

The common opening for the endolymphatic canals of extant chimaeroids is much smaller than the endolymphatic fossa of neoselachians. It forms a small, rounded and not floored fossa on the dorsal surface of the neurocranium. Considering the similar pattern and morphology of the otic capsule and the endolymphatic fossa of the sibyrhynchids and the symmoriiformes “*Cobelodus*”, such a small, rounded and not floored endolymphatic fossa is considered in the present work as primary homologous in *Chimaera*, *Iniopera* and Symmoriiformes.

16. Perilymphatic fenestra absent (0), or present (1).

Perilymphatic fenestrae are paired openings on the chondrified floor of the endolymphatic fossa. They are present in neoselachians, *Tristychius* [21] and *Egertonodus* [34].

17. Dorsal part of the adult otico-occipital fissure open (0), or closed (1). [2, 3, 5, 12, 13].

In modern chondrichthyans, as well as in other gnathostomes, an otico-occipital fissure initially separates the occipital pila from the otic capsules in the embryo [11, 27]. This fissure in neoselachians embryos consists in fact of two distinct but interconnected regions: a dorsal part separates the occipital arch from the otic capsule, while a ventral part (or metotic fissure) separates the otic capsules dorsally and a hypotic lamina ventrally (see below). The dorsal part of the otico-occipital fissure in chondrichthyans and osteichthyans is homologous, since it primitively separates the otic capsules from the occipital pilae [1, 3]. The dorsal part of the otico-occipital fissure remains open in adult in early osteichthyans, *Pucapampella*, *Doliodus*, xenacanths, “ctenacanths” and Symmoriiformes. This arrangement may represent the plesiomorphic condition for chondrichthyans, while the derived condition (closure of the fissure in ontogeny) may unite neoselachians, hybodontiforms, and holocephalans [32].

18. Metotic fissure in adult open (0), or closed (1).

The metotic fissure is absent in modern adult chondrichthyans, but may persist as a ventral otic notch in some fossil chondrichthyans (e.g., *Orthacanthus*, *Tamiobatis*, *Cladodoides*, “*Cobelodus*’, *Akmonistion*). It is represented in modern adult neoselachians by the glossopharyngeal canal after the hypotic lamina becomes fused to the floor of the otic capsule [11]. Although parachordals are present in extant chimaeroids, there is no hypotic lamina, but De Beer [11] argued that the glossopharyngeus and vagus nerve foramina are remnants of the metotic fissure.

Gardiner [23] suggested that the vestibular fontanelle of oteichthyans is homologous to the ventral otic notch of *Orthacanthus* because it is located in both between the otic capsule and the parachordal plate (the hypotic lamina of elasmobranchs is usually regarded as a lateral expansion of the parachordal plate [1, 6]). Nevertheless, Maisey [1] pointed out that “the osteichthyan vestibular fontanelle represents only one part of the primitive gnathostome metotic fissure and corresponds to the embryonic basicapsular fenestra (which may remain open even after the rest of the metotic fissure is closed by the posterior basicapsular commissure [11])”. According to De Beer [11], the basicapsular fenestra of extant osteichthyans is delimited anteriorly by the anterior basicapsular commissure, and posteriorly by the basivestibular commissure, or it may be confluent with the foremost part of the metotic fissure, which transmits the glossopharyngeus nerve and is limited posteriorly by the posterior basicapsular commissure. Nevertheless, the metotic fissure of neoselachians, as well as that of osteichthyans, is delimited by anterior and posterior basicapsular commissures. In osteichthyans, the fissure is represented by the basicapsular fenestra (or vestibular fontanelle) and the foremost portion of the metotic fissure, through which passes the glossopharyngeus nerve. The metotic fissure of neoselachians is probably homologues to that of osteichthyans, because of its similar topographic relations to the otic capsule, the parachordal plate and the glossopharyngeus nerve, but its morphology is slightly different because it is not divided into two parts.

19. Glossopharyngeal nerve crossing the cavity of the auditory capsule, and exiting anterior to metotic fissure, when present, ventrolateral, posteroventral to the otic capsule (0), or floored by the hypotic lamina and exiting through the open metotic fissure (1), or floored by the hypotic lamina and enclosed by a glossopharyngeal canal (2).

In extant holocephalans, as well as in iniopterygians and actinopterygians, the glossopharyngeus nerve runs into a very short canal after passing through the posterior part of the saccular chamber, to reach the ventrolateral (in holocephalans and iniopterygians) [15] or lateral (in actinopterygians) wall of the neurocranium [35]. In addition, in these taxa, there is no evidence of a hypotic lamina [1, 11, 30]. The foramen for the glossopharyngeus nerve is situated posteroventral to the otic capsule, either anterior to the vagus one in holocephalans and iniopterygians, or anterior to the open metotic fissure, where the vagus nerve leaves the neurocranium, in primitive osteichthyans, such as *Mimia*. In *Helodus*, there is apparently no hypotic lamina [8], and the glossopharyngeus nerve reached the ventral surface of the neurocranium in the same way as in extant holocephalans and iniopterygians. Its condition is therefore assumed to be similar.

In *Kawichthys*, there is probably an otico-occipital fissure. Nevertheless, the glossopharyngeus nerve passed into short canal through the otic capsule and emerged in the posterolateral part of the neurocranium, as in osteichthyans.

In neoselachians, *Pucapampella*, *Doliodus*, symmoriiformes, ctenacanths and xenacanths, the glossopharyngeal nerve is floored by the hypotic lamina. The glossopharyngeal canal either exits through the metotic fissure in *Pucapampella*, *Doliodus*, symmoriiformes, “ctenacanths” and xenacanths, or runs into a canal and exits through the remnant of the metotic fissure (neoselachians).

20. Ventral otic fissure in adult absent (0), or present (1). [2, 5, 12].

Osteichthyans, acanthodians and *Pucapampella* possess a persistent ventral otic fissure ventrolaterally, which separates the parachordal plate from the rest of the basicranium. Patterson [8] concluded that otico-occipital and ventral otic fissures of modern osteichthyans have different ontogenies, morphologies and evolutionary histories. In the other taxa considered here, there is no evidence of a ventral otic fissure.

21. Notochord short and stopped by occipital cotylus situated at the rear of the occipital region absent (0), or present (1). [2].

In all modern chondrichthyans except batoids, the embryonic notochord extends between the embryonic parachordal cartilages as far as the posterior margin of the embryonic trabeculae, where it usually reaches the dorsum sellae [11, 27]. In adult elasmobranchs, the cranial part of the notochord is reduced or obliterated, and the fate of the notochord is variable.

In most of fossil chondrichthyans studied here, an occipital cotylus, or basioccipital fovea is present. It encloses a short notochordal canal, which extends only a short distance into the parachordal region and does not reach the dorsum sellae (e.g., *Egertonodus* [4]; *Cladodoides* [1]; “*Cobelodus*” [6]; *Orthacanthus*, *Tamiobatis* [3]; *Akmonistion* [13]; *Iniopera* [15]).

This condition contrasts with that of *Notorynchus* [26] and *Squalus* [11], in which the notochord extends below the otic region between the two embryonic parachordal cartilages. In extant holocephalans, the notochord is free inside the endocranium as far as the dorsum sellae [30], which marks the anterior limit of the embryonic parachordal cartilages.

In *Pucapampella*, the notochord extends forward in the parachordal plate almost as far as its anterior end [2].

In *Mimia*, as in most osteichthyans, the notochordal canal extends forward beneath the otic region as far as the ventral otic fissure and prootic bridge [23]. The latter has been considered as homologues to the dorsum sellae of chondrichthyans.

22. Occipital block wedged between otic capsules (0), or entirely posterior to them (1). [2, 3, 5, 12, 13].

In osteichthyans, as well as in placoderms, *Acanthodes*, *Pucapampella* and *Doliodus*, the occipital region is located posterior to the otic capsules, suggesting that this condition is plesiomorphic for chondrichthyans. In extant chondrichthyans, much of the occipital arch is situated more anteriorly, between the rear of the otic capsule. This condition occurs in other Paleozoic choncrichthyans, even in those whose occipital region extends a considerable distance posteriorly (e.g., *Orthacanthus*, *Tamiobatis* and *Cladodoides*) [32].

23. Hyomandibular articulation on the postorbital process (0), on the otic capsule (1), or not articulated to the braincase (2).

*Helodus* [8] and the Sibyrhynchidae [15] have a holostylic jaw suspension, as in modern holocephalans, which does not involve an articulation between a hyomandibula and the neurocranium. In all the other chondrichthyans of the present analysis, the hyomandibular is attached to the neurocranium on the otic capsule.

In *Tristychius*, in which the suspensorial arrangement is clearly present in several articulated specimens [21], the hyomandibulaa articulates with the neurocranium immediately posterior to the postorbital process, in front of the foramen for the glossopharyngeus nerve. Thereby the hyomandibular articulation occurs in the anterior part of the otic capsule, contrary to most other Paleozoic non holocephalan-like chondrichthyans, hybodonts and orbitostylic sharks.

In actinopterygians and placoderms [3, 6, 23, 24], the hyomandibular usually articulates with the braincase on or adjacent to the inferred position of the lateral commissure.

24. Postorbital articulation absent (0), on the inferred chondrified lateral commissure (1), or on the primary postorbital process (2).

The articular surface of hexanchiforms (e.g., *Notorynchus*) is located on the primary postorbital process [26], which is a small blastemic element situated just in front of the prootic foramen [27]. By contrast, the postorbital articulation in *Pucapampella*, *Doliodus*, Symmoriiformes, xenacanths, “ctenacanths”, which possess a chondrified lateral commissure, is located farther posteroventrolaterally, ventrolateral to the presumed position of the otic lateral line nerve and the jugular vein. In these taxa, the articular surface is assumed to be presumably located on the distal part of the inferred chondrified lateral commissure. The articular surface faces posteriorly and two regions are distinct (an oblique articular ridge forming the upper part of the articular surface, and a deep articular groove below the articular ridge). In addition, in these taxa, the palatoquadrate apparently possesses an expanded postorbital region. A postorbital articulation on the chondrified lateral commissure is also present in some hybodontiforms, such as *Tristychius* [21] and *Acrodus* [36], and in *Acronemus*, a stem euselachians [37].

There is no postorbital articulation in extant chimaeroids, even during the ontogeny [11, 31]. We do not have any embryological information for the holostylic fossil forms, such as *Helodus* and *Iniopera*, even though, despite the presence of laterally expended postorbital in the braincase of the latter, the palatoquadrate of *Iniopera* probably made a connection with rather a basitrabecular process posteriorly during the development of the skull, as in extant chimaeroids, than a lateral commissure [15].

25. Ethmoidal articulation absent (0), or present (1). [2].

According to Maisey [1], an ethmoidal articulation is defined as being situated far anteriorly, on the inferred embryonic trabecula. It is not associated with either the foramen for the efferent pseudobranchial artery, or the inferred anterior margin of the polar catilage.

Such a disposition is found in *Debeerius* [22], *Egertonodus* [4] and *Pucapampella* [20] among the chondrichthyans studied here.

As demonstrated by Maisey [1], this definition precludes the previous assumption that an ethmoidal articulation is present in *Orthacanthus, Tamiobatis* and *Akmonistion*. Their articulation is instead considered homologous to the orbital articulation of orbitostylic sharks, but is located in a more anterior position. Nevertheless, a “rostral articulation” is present in *Orthacanthus* [1, 3], and it may be homologous to the ethmoidal articulation of *Egertonodus* that is apparently quite common in “long-jawed” hybodonts, or to the membranous attachment of the anterior end of the palatoquadrate with the internasal plate of *Chlamydoselachus*.

The condition in *Tristychius* is also uncertain. The palatoquadrate articulates with the anterior part of the braincase [21], but there is no evidence of a foramen for the efferent pseudobranchial artery. Consequently, this articulation may be either an ethmoidal articulation, or an orbital articulation, which is located far anteriorly, as in *Orthacanthus* and *Cladodoides*.

Grogan et al. [31] observed that the palatoquadrate of extant chimaeroids first meets the ethmoidal plate early in development. In addition, this contact apparently occurs far anteriorly from the foramen for the efferent pseudobranchial artery, suggesting that an ethmoidal articulation is present in this taxon, at least in embryos. Grogan and Lund [22] also described an ethmoidal articulation and a palatobasal (or orbital, see below) articulation in the autodiastylic stem-holocephalan *Debeerius*. These observations suggest that an ethmoidal articulation was primitively present in holocephalans. In *Iniopera* and *Helodus*, which also have a holostylic jaw suspension, the condition was probably the same as in extant chimaeroids, but no embryological data are available.

In actinopterygians, an “autopalatine” or “ethmoid” articulation is present (e.g. *Mimia* [23]). Following Maisey [1], it is considered in the present work as being homologues to the ethmoidal articulation of chondrichthyans, since it is developed on the lateral margin of the inferred embryonic trabecula, and involves the anteriormost part of the palatoquadrate. They also have a palatobasal articulation.

26. Orbital (or palatobasal) articulation absent (0), anterior to the optic foramen (1), or posterior to the optic foramen (2). [5].

Maisey (2005) discussed the question of homology between the previously presumed ethmoidal articulation of extinct chondrichthyans such as *Orthacanthus*, *Tamiobatis* [3] and *Akmonistion* [13] and the orbital articulation of living orbitostylic sharks. In both case, there is the same relationship to landmark arterial foramina and presumably to the polar cartilage. The main difference is that in these Paleozoic taxa, the articulation occurs anterior to the optic foramen. The anterior part of the inferred polar cartilage of *Kawichthys* flares laterally (see above). It is assumed that it corresponds to an “anterior” orbital articulation, as present in the fossil chondrichthyans mentioned above.

In *Tristychius,* assuming that the anterior articulation observed by Dick [21] is actually an orbital articulation, it is probably located anterior to the optic nerve, since it is situated at the anterior margin of the orbit, and the optic nerve is usually located at mid-length of the orbit in chondrichthyans.

In osteichthyans, orbitostylic neoselachians [38], *Debeerius* and *Pucapampella*, the basitrabecular process is situated posterior to the optic foramen. In “*Cobelodus*”, the articular surface of the neurocranium seems to be situated posterior to the optic nerve [6], which is a different condition from other Paleozoic taxa, such as *Orthacanthus*, or *Akmonistion*.

In modern chimaeroid embryos, the palatoquadrate first attaches to the basitrabecular process, which is located posterior to the optic nerve [31]. In *Iniopera*, the foramen for the efferent pseudobranchial artery, which in modern elasmobranchs delimits the anterior margin of the polar cartilage (hence the basitrabecular process), is situated posterior to the optic foramen [15]. Consequently, if a palatobasal articulation formed during the ontogeny of Sibyrhynchidae, it should be located behind the optic foramen.

27. Orbital articulation extending upward into the orbit absent (0), or present (1).

As mentioned above, the orbital articular surface of “*Cobelodus*” differs from that of other Paleozoic taxa in its posterior position relative to the optic foramen, as in modern orbitostylic neoselachians. Nevertheless, it differs from the latter in its entirely ventral position, below the optic foramen, as in osteichthyans, stem-holocephalans, other symmoriiforms, “ctenacanths” and xenacanths. By contrast, in modern orbitstylic neoselachians, the dorsal margin of the palatoquadrate is produced into an orbital process that extends a considerable distance into the orbit and is housed inside a vertical groove on the basitrabecular process [26].

28. Internal carotid artery supplying the blood to the head absent (0), or present (1).

In gnathostomes generally, the internal carotid arteries supply the blood to the brain.

In *Tamiobatis* sp., there is no grooves for the internal carotid arteries. Nevertheless, the slabbed specimen (AMNH 2140) displays the internal course of internal carotid arteries from the ventral surface to the cranial cavity, behind the bucco-hypophyseal fenestra [1, 3].

In *Kawichthys*, there are also no grooves for the internal carotid arteries. Nevertheless, the lateral dorsal aortae ran through canals within the basicranium. The canals seem to continue anteriorly after their meeting with the orbital and hyoid arteries, suggesting that the internal carotid arteries supply blood to the brain. However, there is no clear evidence for that, since the endocranial cavity is missing at the level of the hypophyseal region.

The condition is different in extant holocephalans. There are no grooves for internal carotid arteries, and the latter never supply blood to the brain in adults [30], even if they may enter the basicranium in some species [29]. Even though there is neither a pseudobranch, nor a spiracle in the adults, an uninterrupted efferent “pseudobranchial” artery is present on each side of the neurocranium, which ensures the only blood supply to the brain via the orbits. This condition is considered to be similar in the iniopterygians studied here, since there is no evidence of grooves or foramina for the internal carotid arteries.

In *Helodus*, a holostylic chondrichthyan, like extant chimaeroids, there is a “ventral short aortic canal, which is continued forward as a shallow groove almost to the hypophysial foramen” [7]. This groove is assumed to have housed the dorsal aorta. According to Maisey [6], a dorsal aorta also extends beneath the basicranium almost until the bucco-hypophyseal fenestra in the squaloid *Aculeola nigra*. Then, it divides into short lateral aortae that meet the internal carotid and efferent hyoidean arteries. Such a condition may have occurred in *Helodus*, although there is no evidence of grooves for internal carotid arteries. Consequently, its dorsal aorta may have divided into lateral dorsal aortae, but not internal carotid arteries, as in most extant chimaeroids. Therefore, the bucco-hypophysial foramen may not have housed the internal carotid arteries, as in extant chimaeroids. One may also suggest that, as in *Rhinochimaera* [29], the internal carotid arteries entered the skull through an unpaired foramen, but did not supply the blood to the brain.

In *Pucapampella*, the condition remains problematic to date. The South African specimen [20] does not possess grooves for internal carotid arteries but there is a median, bilaterally symmetrical opening with a central constriction. This suggests that this opening housed both the internal carotid arteries and the bucco-hypophyseal duct. It was previously assumed by Maisey [2] that the pair of smaller, widely-spaced basicranial foramina housed the internal carotid arteries in the Bolivian specimen. Nevertheless, these foramina are also present in the South African braincase, leading Maisey and Anderson [20] to reconsider the condition in the Bolivian braincase. They assumed that the paired foramina contained the efferent pseudobranchial arteries. A pair of median, bilaterally symmetrical openings may be also present in this specimen. A re-evaluation of the specimen will be provided in further studies.

29. Dorsal aorta divides into lateral dorsal aortae anterior to the occipital region (0), or posterior to the occipital region (1). [2, 13].

The presence of a dorsal aortic canal or groove suggests that the lateral dorsal aortae diverged from the dorsal aorta anterior to the level of the occiput. Among Paleozoic chondrichthyans, this arrangement is also present in *Akmonistion* [13], “*Cobelodus*” [6], *Cladoselache* [6], *Kawichthys* and *Helodus* [7, 8]. In extant chimaeroids, there is neither a dorsal aortic canal, nor a dorsal aortic groove. Nevertheless, according to De Beer and Moy-Thomas [30], the dorsal aorta of the adult chimaeroids divides into lateral dorsal aortae below the foramen for the vagus nerve, and neither the dorsal aorta nor the lateral dorsal aortae enter the basicranium through a canal or run into grooves on the ventral surface of the basicranium. In neoselachian, such as *Notorynchus* [26] and *Squalus* [39], there is also neither a dorsal aortic canal, nor a dorsal aortic groove, and there are also no occipital foramina for the lateral dorsal aortae. The dorsal aorta separates into paired lateral dorsal aorta when it reaches the hind end of the ventral surface of the neurocranium.

A dorsal aortic canal is present in *Mimia* [23].

The other Paleozoic taxa considered in the present analysis display yet another condition. There is neither a dorsal aortic canal, nor a dorsal aortic groove, and the lateral dorsal aortae probably diverged from the dorsal aorta posterior to the occipital level. This is illustrated by the presence of paired foramina for the lateral dorsal aortae in the posterior part of the occipital region, as in *Orthacanthus*, *Tamiobatis* [3], *Tristychius* [21], *Ergetonodus* [4], *Cladodoides* [1], *Pucapampella* [20] and *Doliodus* [19].

30. Dorsal aorta free (0), into a canal (1), or into a deep groove (2).

We have seen above that neoselachians, chimaeroids and symmoriiformes possess a dorsal aorta which divides into lateral dorsal aortae anterior to the occipital region. Nevertheless, only the latter group display a dorsal aorta canal. *Kawichthys* possesses a deep groove for the dorsal aorta.

31. Lateral dorsal aorta canals absent (0), or present (1). [3, 5, 12, 13].

Among chondrichthyans, the lateral dorsal aortae are either free underneath the basicranium, or enclosed inside canals. The lateral dorsal aortae are free underneath the basicranium in neoselachians and extant chimaeroids. Patterson [8] and Coates and Sequeira [13] also claimed that *Helodus* does not possess lateral aortic canals, and it is assumed that *Iniopera* [15], as well as *Tristychius* [21], also possessed free lateral dorsal aortae. In *Egertonodus*, the condition is not clear. Maisey [4] considered that *Egertonodus* had merely very short lateral aortic canals, which may represent, according to Coates and Sequeira [13], an autapomorphy or a “transitional state between canal presence and absence”. In fact, a new investigation found no evidence of a canal along the course of the dorsal aorta in *Egertonodus* [40]. In this taxon, as well as in *Tribodus*, neoselachians, chimaeroid embryos, probably *Iniopera*, *Helodus* and *Tristychius*, the lateral dorsal aortae seem to have divided into internal carotid arteries well outside the neurocranium, since grooves for internal carotid arteries are visible at the level where the lateral dorsal aortae are not enclosed into canals.

32. Distinct foramina for hyoidean artery absent (0), or present (1). [2].

*Kawichthys* possesses a pair of distinct efferent hyoidean foramina, through which the efferent hyoidean arteries passed to meet the lateral dorsal aortae. Coates and Sequeira [13] supposed that *Akmonistion* also possesses this condition, although Maisey [1] argued that these formina may be for the palatine ramus of the facial nerve. Nevertheless, the basicranial foramina of *Kawichthys* and *Akmonistion* are very similar, and here is accepted the Coates and Sequeira’s [13] interpretation. The condition in *Tamiobatis* is doubtful. Schaeffer [3] considered that this taxon possesses distinct foramina for the the hyoidean arteries. By contrast, the comparison of the vascular and nerve pattern of *Cladodoides* and *Orthacanthus* leads Maisey [1] to interpret these foramina as openings for the palatine ramus of the facial nerve.

33. Unchondrified medial capsular wall absent (0), or present (1). [3].

In Recent elasmobranchs, the endocranial and labyrinth cavities are almost completely separated from each other by a medial capsular wall, which is well chondrified, apart from the foramina for the octaval and glossopharyngeus nerves and the perilymphatic fenestra. The medial capsular wall forms mainly in the taenia medialis, which also floors the endolymphatic fossa [11, 27].

Such a condition is also present in hybodonts, such as *Egertonodus* [33] and *Tribodus* [40]. It is also probably present in *Tristychius*, since, according to Maisey [1], “Dick (1978, figs. 2, 4) showed extensive chondrification on each side of the “median supraotic fossa” extending into the braincase medial to the otic capsule”. In addition, the endolymphatic ducts and perilymphatic fenestrae reach the “median supraotic fossa”, which is floored as in neoselachians. Consequently a taenia medialis was probably present.

By contrast, the medial capsular wall is unchondrified in many Paleozoic chondrichthyans (e.g., *Pucapampella*, *Cladodoides*, “*Cobelodus*”, *Orthacanthus*, *Tamiobatis*, *Iniopera* and *Kawichthys*), as well as in osteichthyans and extant chimaeroids. According to Maisey [1], this is probably the consequence of an absence of chondrification in the taenia medialis. This is supported by the fact that taxa which do not possess a chondrified medial capsular wall, also do not display a floor of the endolymphatic fossa.

34. Crus commune between anterior and posterior semicircular canals absent (0), or present (1). [3].

In extant lampreys, osteichthyans and holocephalans, the anterior and posterior canals meet dorsally to form a crus commune. By contrast, in modern elasmobranchs, the anterior and horizontal semicircular canals are connected together [11, 32, 34]. The posterior semicircular canal appears thus separated to form a complete circuit, and is connected only to the vestibular region.

The former condition seems to be plesiomorphic, since osteostracans, galeaspids [41], early osteichthyans (e.g., *Mimia* [27]), acanthodians [25] and many Paleozoic chondrichthyans, such as *Pucapampella* [2, 33], *Cladodoides* [1], “*Cobelodus*” [6], *Tristychius* [21] and iniopterygians [15] display much the same arrangement. Among the fossils studied here, only *Egertonodus* possesses the latter arrangement [32, 33], but it was also been present in *Synechodus*.

Schaeffer [3] suggested that the skeletal labyrinth in *Orthacanthus* display a similar arrangement as in neoselachians, i.e. the posterior semicircular canal forms an almost complete circuit, so that a crus commune is present between the horizontal and anterior semicircular canals. Nevertheless, according to Maisey [1, 33], it is doubtful that the posterior semicircular canal possesses an extensive ascending pre-ampullary region. Moreover, the dorsal part of the anterior and posterior semicircular canals are situated close together, so that a crus commune, which connected them, was probably present. It is thus assumed here that this taxon displays the same arrangement as that of osteichthyans, acanthodians and some of the Paleozoic chondrichthyans cited above. The condition in *Tamiobatis* sp. is also considered similar by Maisey [33].

**References**

[1] Maisey JG (2005) Braincase of the Upper Devonian shark *Cladodoides wildungensis* (Chondrichthyes, Elasmobranchii), with observations on the braincase in early chondrichthyans. B Am Mus Nat Hist 288: 1-103.

[2] Maisey JG (2001) A primitive chondrichthyan braincase from the Middle Devonian of Bolivia. In: Ahlberg PE, editor. Major events in early vertebrate evolution: paleontology, phylogeny, genetics and development. New York: Taylor and Francis for the Systematics Association. pp. 263-288.

[3] Schaeffer B (1981). The xenacanth shark neurocranium, with comments on elasmobranch monophyly. B Am Mus Nat Hist 169: 3-66.

[4] Maisey JG (1983) Cranial anatomy of *Hybodus basanus* Egerton from the Lower Cretaceous of England. Amer Mus Novitates 2758: 1-64.

[5] Brazeau M. (2009) The braincase and jaws of a Devonian ‘acanthodian’ and modern gnathostome origins. Nature 457: 305-308.

[6] Maisey JG (2007) The braincase in Paleozoic symmoriiform and cladoselachian sharks. B Am Mus Nat Hist 307: 1-122.

[7] Moy-Thomas JA (1936) On the structure and affinity of the Carboniferous cochliodont *Helodus simplex.* Geol Mag 73: 488-503.

[8] Patterson C (1965) The phylogeny of the chimaeroids. Philos T R Soc Lon B 249: 101-219.

[9] Williams ME (1985) The ‘‘cladodont level’’ sharks of the Pennsylvanian black shales of central North America. Palaeontogr Abt A 190: 83-158.

[10] Lund R, Grogan ED (1997) Relationships of the Chimaeriformes and the basal radiation of the chondrichthyes. Rev Fish Biol Fisher 7: 65-123.

[11] De Beer GR (1937) The development of the vertebrate skull. Oxford: Clarendon Press. 552 p.

[12] Coates MI, Sequeira SEK (2001) Early sharks and primitive gnathostome interrelationships. In: Ahlberg PE, editor. Major events in early vertebrate evolution: paleontology, phylogeny, genetics and development. New York: Taylor and Francis for the Systematics Association. pp. 241-262.

[13] Coates MI, Sequeira SEK (1998) The braincase of a primitive shark. T Roy Soc Edin-Earth 89: 63-85.

[14] Maisey JG (1984) Chondrichthyan phylogeny: a look at the evidence. J Vert Paleont 4(3): 359-371.

**[15] Pradel A (2010) Skull and brain anatomy of Late Carboniferous Sibyrhynchidae (Chondrichthyes, Iniopterygia) from Kansas and Oklahoma (USA). Geodiversitas 32(4): 595-661.**

[16] Maisey JG (1985) Cranial Morphology of the fossil elasmobranch *Synechodus dubrisiensis*. Amer Mus Novitates 2804: 1-28.

[17] Jarvik E (1980) Basic structure and evolution of vertebrates. London: Academic Press. 575 p.

[18] Didier D (1995) Phylogenetic systematics of extant chimaeroid fishes (Holocephali, Chimaeroidei). Amer Mus Novitates 3119: 1-86.

[19] Maisey JG, Miller R, Turner S (2009) The braincase of the chondrichthyan *Doliodus* from the Lower Devonian Campbellton formation of New Brunswick, Canada. Acta Zool 90(Suppl.1): 109-122.

[20] Maisey JG, Anderson ME (2001) A primitive chondrichthyan braincase from the early Devonian of South Africa. J Vert Paleont 21(4): 702-713.

[21] Dick JRF (1978) On the Carboniferous shark *Tristychius arcuatus* Agassiz from Scotland. T Roy Soc Edin 70: 63-109.

[22] Grogan ED, Lund R (2000) *Debeerius ellefseni* (Fam. nov., Gen. nov., Spec. nov.), an autodiastylic chondrichthyan from the Mississippian Bear Gulch Limestone of Montana (USA), the relationships of the Chondrichthyes, and comments on gnathostome evolution. J Morphol 243: 219-245.

[23] Gardiner BG (1984) The relationships of the palaeoniscid fishes, a review based on new specimens of *Mimia* and *Moythomasia* from the Upper Devonian of Western Australia. Bul Br Mus (Nat Hist) Geol 37(4): 173-428.

[24] Goujet D (1984) Les poissons Placodermes du Spitzberg. Arthrodires Dolichothoraci de la Formation de Wood Bay (Dévonien Inferieur). Paris: Cahiers de Paléontologie, Centre National de la Recherche Scientifique. 284 p.

[25] Miles RS (1973) Relationships of acanthodians. In: Greenwood PH, Miles RS, Patterson C, editors. Interrelationships of fishes. London: Academic Press. pp. 63-103.

[26] Maisey JG (2004) Morphology of the braincase in the broadnose sevengill shark *Notorynchus* (Elasmobranchii, Hexanchiformes), based on CT scanning. Amer Mus Novitates 3351: 1-2.

[27] Holmgren N (1940) Studies on the head in fishes. Part I. Development of the skull in sharks and rays. Acta Zool-Stockholm 21: 51-67.

[28] Maisey JG (2008) The postorbital palatoquadrate articulation in Elasmobranchs. J Morphol 269: 1022-1040.

[29] Holmgren N (1942) Studies on the head in fishes. Part III. The phylogeny of elasmobranch fishes. Acta Zool-Stockholm 23: 129-261.

[30] De Beer GR, Moy-Thomas JA (1935) On the skull of Holocephali. Philos T R Soc Lon B 224: 287-12.

[31] Grogan ED, Lund R, Didier D (1999) Description of the Chimaerid Jaw and its Phylogenetic Origins. J Morphol 239: 45-59.

[32] Maisey JG, Lane JA (2010) Labyrinth morphology and the evolution of low-frequency phonoreception in elasmobranchs. CR Palevol 9(2010): 289-309.

[33] Maisey JG (2004) Endocranial morphology in fossil and Recent chondrichthyans. In: Arratia G, Wilson M, Cloutier R, editors. Recent advances in the origin and radiation ofearly vertebrates. Munich: Pfeil. Pp. 139-170.

[34] Maisey JG (2001) Remarks on the inner ear of elasmobranchs and its interpretation from skeletal labyrinth morphology. J Morphol 250: 236-264.

[35] Cole FJ (1896) On the cranial nerves of *Chimaera monstrosa* (Linn. 1754); with a discussion of the lateral line system, and of the morphology of the chorda tympani. T Roy Soc Edin 38: 49-56.

[36] Thomson KS (1982) An early Triassic hybodont shark from northern Madagascar. Postilla 180: 1-16.

[37] Maisey JG (2011) The braincase of the Middle Triassic shark *Acronemus tuberculatus* (Bassani, 1886). Palaeontology 54(2): 417-428.

[38] Maisey JG (1981) An evaluation of jaw suspension in sharks. Amer Mus Novitates 2706: 1-17.

[39] Marinelli W, Strenger A (1959) Vergleichende Anatomie und Morphologie der Wirbelthiere. III. Lieferung, pt. 2, *Squalus acanthias*. Vienna: Franz Deuticke. pp. 179-308.

[40] Lane JA (2010) Morphology of the braincase in the Cretaceous hybodont shark Tribodus limae (Chondrichthyes: Elasmobranchii), based on CT Scanning. Amer Mus Novitates 3681: 1-70.

[41] Janvier P (1996) Early vertebrates. Oxford: Clarendon Press. 392 p.
