# Supplementary material for: A New Paleozoic Symmoriiformes (Chondrichthyes) from the Late Carboniferous of Kansas (USA) and Cladistic Analysis of Early Chondrichthyans
Source: PLoS One. 2011 Sep 27;6(9):e24938. doi: 10.1371/journal.pone.0024938 (PMC3181253; doi:10.1371/journal.pone.0024938)
Supplement: Text S2 — Data Matrix used for the phylogenetic analysis. (DOC) [file pone.0024938.s002.doc]

**Text S2 – Data Matrix used for the phylogenetic analysis**

| 0 10 20 30 |
| --- |

*Akmonistion* 0?0?10?10? 001?11?001 0111101010 111??

*Chimaera* 0010100000 0000110110 0012012000 00011

*Cladodoides* 000110010? 0010100001 0111101011 01011

*Cladoselache* 0?0?10?100 01?????0?? 0?1?10?010 110??

“*Cobelodus*” 0101100100 0100110001 0111102010 11011

*Debeerius* 0?0010?000 000?11?11? 00?201201? 000?1

*Doliodus* 00011??100 000000?0?1 0?01101011 010?1

*Egertonodus* 0001111100 0001101112 0111010011 0?000

*Helodus* 0010100000 00?????110 0?12???0?0 100??

*Iniopera* sp. 0010100100 0001110110 0112???00? 00011

*Kawichthys* 010?10?100 0001110??0 00111010?0 21111

*Notorynchus* 0001010210 1001101112 0011202111 00000

*Orthacanthus* 1001100101 0010100001 01111?1011 01011

*Pucapampella* 000?100100 0000000001 10011120?1 01011

*Squalus* 0001010210 1001101112 0011002111 00000

*Synechodus* 0001000200 000110?112 0?1121001? 000??

*Tamiobatis* sp. 1001100101 0010100001 0111101011 01?11

*Tristychius* 0001111100 0001101112 0?111??011 0000?

*Mimia* 0100100100 0000000000 1000012010 11011
